# Interacting Parallel Fluidic Hysterons

**DOI:** 10.1002/advs.76158

**Published:** 2026-06-25

**Authors:** Katrien Stinissen, Franco Nicolas Piñan Basualdo, Benjamin Gorissen

**Affiliations:** ^1^ Department of Mechanical Engineering KU Leuven Leuven Belgium

**Keywords:** fluidic coupling, hysterons, inflatables, physical intelligence

## Abstract

Structures exhibiting highly nonlinear behavior are essential for realizing advanced functionality without software; however, their nonlinearity gives rise to complex and difficult‐to‐predict interactions. Among these nonlinear structures, fluidic hysterons — nonlinear fluidic elements that retain memory — stand out as particularly promising for realizing complex behaviors in inflatable soft systems. To date, inflatable hysteretic elements have been almost exclusively coupled in pressure‐shared configurations, corresponding to a series connection in an energetic sense. Here, a complementary equal‐volume‐change architecture is studied, in which inflatable hysterons are coupled in parallel. This study examines the principles governing how nonlinear inflatable structures, such as fluidic hysterons, behave when connected in pressure‐volume space. A general framework is developed to describe both series and parallel connections, and a practical strategy based on presetting pressures and volumes is introduced to alter the interactions. Experiments on parallel‐connected fluidic hysterons validate the analytical predictions and demonstrate how preset volume provides a practical control parameter for tuning interaction strength and functional response. These results establish volume‐constrained parallel coupling as a new design principle for inflatable systems with distributed memory and embodied computation.

## Graphical abstract

The parallel coupling of fluidic hysterons is introduced, establishing advanced functionalities in inflatable soft systems. A pressure–volume framework reveals how preset volumes $\Delta v^{*}$ tune nonlinear interactions between hysterons and actuation sequences without changing architecture. Experiments validate the predictions, opening new routes toward programmable pneumatic networks that compute through structure and mechanics.

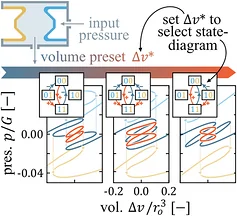

Intelligent behavior emerges when sensing, actuation, and computation are integrated within a single system that can perform tasks in a perceived environment [1]. Traditionally, robots rely on software‐based computation to encode control and decision‐making. However, this approach can be limited in unstructured or dynamic settings due to the separation between the computing units and the physical hardware. To address this issue, researchers have partially offloaded computation onto the body itself, a concept known as morphological computation [2], which enables more robust and adaptable behavior. More recently, computation has been embedded directly into the hardware, exploiting the interaction between nonlinear physical elements [3, 4, 5, 6, 7]. This software‐free paradigm distributes computation across the physical structure, enabling decentralized information processing, leading to systems capable of adapting to changing environments through their morphology alone.

Networks of inflatables offer a compelling example of software‐free intelligence. The nonlinear behavior of inflatable networks leads to system‐level behaviors that emerge purely from physical interactions. Nonlinearities may arise from the interconnections between the inflatable structures [8, 9, 10] or from the fluids within them [11, 12]. In most cases, however, it is the nonlinear response of the inflatable structures themselves that is exploited for functional purposes [13, 14, 15, 16]. Leveraging these nonlinear characteristics can significantly enrich the behaviors of inflatable networks while simplifying their actuation [17].

An interesting class of inflatable component is the *fluidic hysteron* [13], which exhibits nonlinear hysteretic behavior analogous to the path‐dependent hysteresis loops of ferromagnetic materials [18]. These hysteretic components, which are found across multiple physical domains [19, 20, 21, 22, 23], exhibit outputs that depends on the input history, enabling their use as memory elements [24]. In mechanical systems, bistable structures provide history‐dependent responses that are exploited for programmable mechanics [25], energy absorption [26], and controlled transitions [27]. In inflatable systems, hysteretic behavior can occur at the component level [4, 13] or emerge from the interaction between interconnected components [15, 28].

While individual fluidic components can yield interesting behaviors, networks of interacting hysterons can achieve advanced system‐level functionality. Two fundamental interconnection topologies exist for inflatable systems, distinguished not by geometric layout or shared manifolds, but by how pressure and volume are coupled across elements. In a series connection, inflatable components are subjected to the same internal pressure and their volumes add; in a parallel connection, components undergo the same volume change and their pressures add. This definition mirrors the classical distinction between series and parallel connections of mechanical springs and hydraulic pistons and is consistent with established treatments of serial inflatable systems [15, 29]. Importantly, most inflatable networks studied in soft robotics, such as multi‐chamber actuators supplied by a common pressure source, fall into the series category under this energetic definition, even when chambers are geometrically arranged in parallel [30]. By contrast, architectures that enforce equal volume change across multiple inflatables, thereby producing pressure‐adding interactions, have received little attention.

Building on these insights, we introduce a model system of parallel inflatable fluidic hysterons, highlighting the duality with serial systems. Additionally, we propose internal volume as a preset control parameter that allows tuning of the system's inflation response in real time, without altering the physical structure, connectivity, or loading conditions [13, 15]. The complex response of two parallel fluidic hysterons is modeled using a reduced‐order approach, extending the theory of Liu et al. [27]. As a result, we derive an analytical solution that accurately predicts the system's collective and local behavior during generalized loading. This modeling framework is validated through experiments on parallel connections of fluidic hysterons, paving the way for designing intricate fluidic networks with arbitrary connectivity and functional richness.

## 1 Results

### 1.1 Connecting Inflatable Structures

Inflatables deform in response to internal pressure and are typically characterized by a pressure–volume (pv) curve, which captures the relationship between internal pressure and enclosed volume at equilibrium. The shape of an inflatable's pv‐curve can vary widely, from monotonic where there is a direct relation between pressure and volume [31], to multivalued where a single pressure can correspond to multiple stable volumes, and a single internal volume can be stable at different pressure levels [32]. When such elements are fluidically connected, their individual responses alone are insufficient to predict the behavior of the combined system; instead, the coupling mechanism between pressure and volume determines the nature of the interactions [15]. In what follows, two inflatable structures with individual pressure–volume relations $p_{1}=f_{1}\left(\Delta v_{1}\right)$ and $p_{2}=f_{2}\left(\Delta v_{2}\right)$ are considered. Two fundamental coupling architectures arise, defined by which variable, pressure or volume, is shared across the system.

**FIGURE 1:**
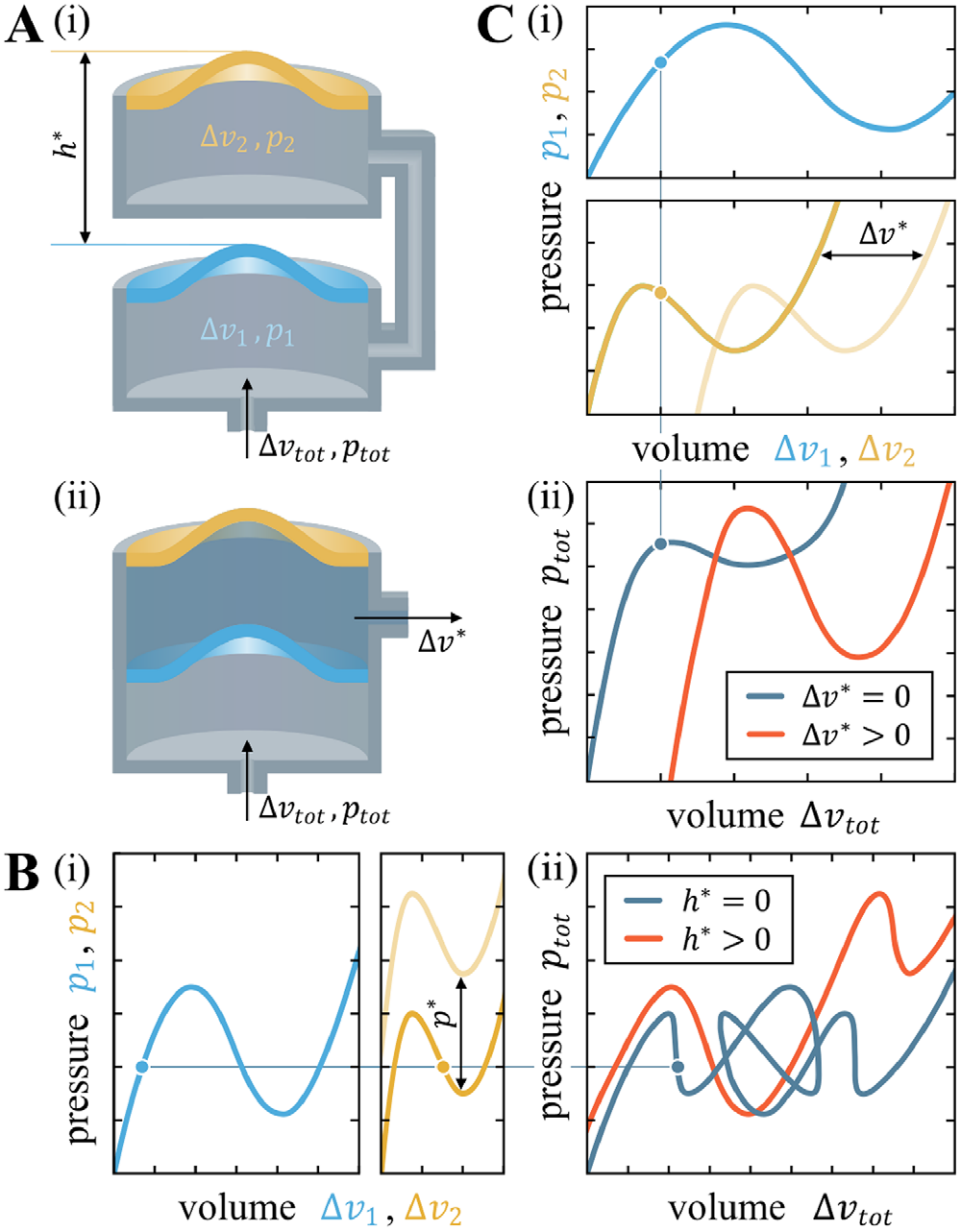
Fluidic Connections of Inflatable Structures in Series and Parallel. (A) Schematic of two inflatable structures characterized by known pressure–volume (pv) curves $p_{1}=f_{1}\left(\Delta v_{1}\right)$ and $p_{2}=f_{2}\left(\Delta v_{2}\right)$, connected in series (i) and parallel (ii). In the series configuration, a hydrostatic pressure preset $p^{*}=\rho gh^{*}$ is applied via a vertical height difference $h^{*}$. The parallel configuration requires an incompressible fluid between the structures to ensure equal volume changes, and a volume preset $\Delta v^{*}$ can be introduced by removing volume from the cavity between the inflatable structures. (B) Construction of the total pv‐curve for the series connection by horizontally adding the pv‐curves of the individual structures. The individual curves (i) can be vertically translated by $p^{*}$ to reflect the hydrostatic pressure preset. This results in a modified total curve (ii, $h^{*}>0$) for the series connection. (C) Construction of the total pv‐curve for the parallel connection by vertically adding the pv‐curves of the individual structures. The individual curves (i) can be horizontally translated by a volume preset $\Delta v^{*}$, resulting in a modified total curve (ii, $\Delta v^{*}>0$) for the parallel connection.

A general representation of two inflatable structures that are **serially connected** is shown in Figure 1A(i). In a series connection, components are subjected to the same internal pressure, while the total volume change is distributed across the individual elements. The governing equations are:

(1) $$\left\{\begin{array}{c} p_{\mathrm{tot}}=p_{1}=p_{2}\\ \Delta v_{\mathrm{tot}}=\Delta v_{1}+\Delta v_{2} \end{array}\right.$$

Volume is referred to as the coupled variable, as it is distributed between the inflatables, and any change in one component's volume affects the others. Pressure, on the other hand, is the uncoupled variable, as it is imposed uniformly across the system and does not vary between structures. Using Equation (1), the total pressure–volume curve can be constructed by horizontally adding the volumes of the structures at each pressure level, as shown in Figure 1B. This pressure‐shared configuration underlies most inflatable networks studied in soft robotics, including multi‐chamber actuators supplied by a common pressure source [14, 15, 17, 30, 33, 34].

By contrast, a parallel connection, illustrated in Figure 1A(ii), enforces equal volume change across elements by coupling them through an incompressible intermediate fluid:

(2) $$\left\{\begin{array}{c} p_{\mathrm{tot}}=p_{1}+p_{2}\\ \Delta v_{\mathrm{tot}}=\Delta v_{1}=\Delta v_{2} \end{array}\right.$$

where $p_{i}$ denotes the pressure difference across inflatable i for a given volume change $\Delta v_{i}$, as characterized by its individual pv‐curve. In this configuration, pressure is the coupled variable, as it is distributed between inflatables, and any change in one's pressure will directly affect the others. Volume is the uncoupled variable, as it is transmitted from one structure to the next and does not vary between structures. Equation (2) can be used to construct the total pv‐curve by vertically adding the pressures of the individual structures for the same volume change as illustrated in Figure 1C.

To actuate an inflatable system, either a pressure (pressure control) or a volume (volume control) can be imposed. In the context of interconnected systems, the type of actuation defines the interaction between the components. When the system is actuated using the uncoupled variable (pressure in a series connection or volume in a parallel connection), the inflatables are decoupled, and their individual responses can be superimposed to predict the total system behavior. In contrast, when the coupled variable is used as the actuation input to the system (total volume in a series connection or total pressure in a parallel connection), interaction between inflatables is promoted as the distribution of the input is not fixed. This interaction leads to emergent system behavior that deviates from the addition of individual responses. These insights brings forward a conceptual analogy: actuating a series configuration with volume control is equivalent to actuating a parallel configuration with pressure control, as in both cases the coupled variable is employed as an input. This equivalence is further illustrated in Section S3.1. Understanding this relationship is crucial for designing fluidic systems where interaction between components is either desired for coordinated functioning or needs to be minimized for intuitive operation.

### 1.2 Presetting Inflatable Connections

The complex behaviors that arise from connecting inflatables are not only due to the nonlinearity of the individual inflatables, but also because of the interactions between them. The richness of interactions is determined by the relative positions of limit points (minima or maxima in pressure and volume) of the individual pv‐curves. For instance, in a series connection, if pressure peaks and valleys lie within a similar range, the global pv‐curve becomes more complex, leading to more interactions between the inflatables and making the resulting behavior of the system harder to predict (Figure 1B). To modify the relative positions of such optima, and thus to vary the system's complexity and functionality, the inflatables can be preset by imposing a difference in the uncoupled variable (pressure $p^{*}$ or volume change $\Delta v^{*}$ for the respective series and parallel cases). For serial connections, this results in an extension of the governing equations:

(3) $$\left\{\begin{array}{c} p_{\mathrm{tot}}=p_{1}=p_{2}+p^{*}\\ \Delta v_{\mathrm{tot}}=\Delta v_{1}+\Delta v_{2} \end{array}\right.$$

where $p^{*}$ represents the pressure preset. As shown in Figure 1B, presetting in a series system results in a relative vertical translation of the pv‐curve. This can be practically achieved by actuating the structures with an incompressible fluid and placing them at different heights ($h^{*}$) as shown in Figure 1A(i). This height difference gives rise to a pressure difference $p^{*}=\rho \;g\;h^{*}$ (with ρ the fluid density and g the gravitational acceleration). In the parallel case, presetting is described by:

(4) $$\left\{\begin{array}{c} p_{\mathrm{tot}}=p_{1}+p_{2}\\ \Delta v_{\mathrm{tot}}=\Delta v_{1}=\Delta v_{2}+\Delta v^{*} \end{array}\right.$$

where volume presetting ($\Delta v^{*}$) results in horizontal translation of the pv‐curve, as shown in Figure 1C. Practically, volume presetting can be imposed by altering the volume of the incompressible fluid between the inflatable structures, as shown in Figure 1A(ii).

In the context of inflatable networks, modifying internal volumes is generally simpler in practice than introducing large spatial offsets between physical components. This suggests that exploring the implementation of inflatable structures in parallel is worthwhile.

Overall, the analogy between series and parallel inflatable systems extends to the concept of presetting. In both cases, the level of interaction can be tuned by adjusting the uncoupled variable: a pressure offset $p^{*}$ in series configurations and a volume offset $\Delta v^{*}$ in parallel configurations.

### 1.3 Parallel Connection of Fluidic Hysterons

**FIGURE 2:**
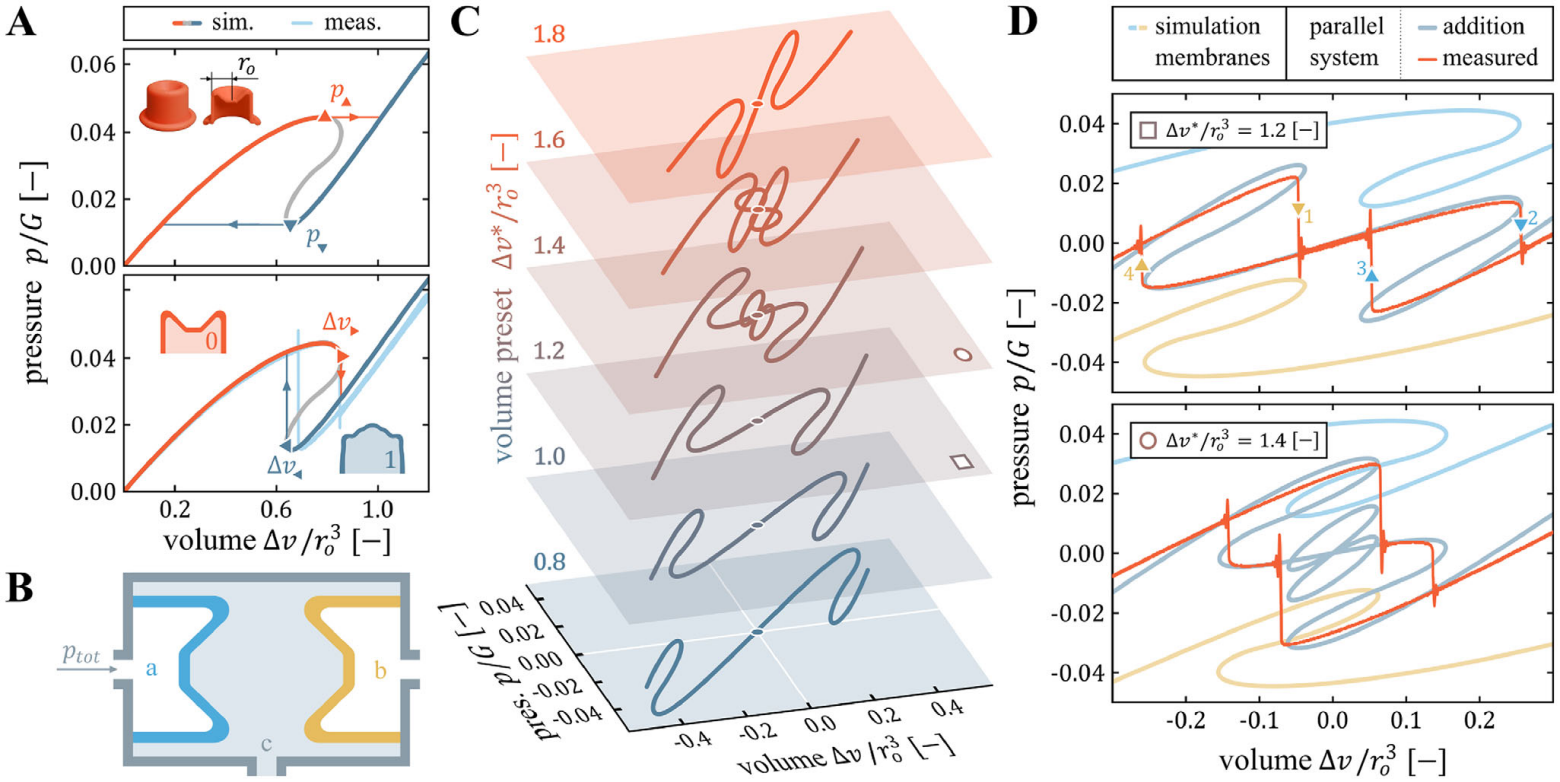
Parallel‐Connected Fluidic Hysterons. (A) Response of the truncated conical membrane used in the parallel connection and its geometry (inset, base radius $r_{o}$). The membrane exhibits the characteristic behavior of a fluidic hysteron, as shown by its pv response under both pressure control (top) and volume control (bottom), as obtained from FEM simulations. The pv‐curves are normalized using the shear modulus G of the material and the base radius $r_{o}$. The membrane can remain stable in either the 0 (down) or 1 (up) state, with transitions governed by the input conditions: $p_{▴}$ and $p_{▾}$ under pressure control, and $\Delta v_{▸}$ and $\Delta v_{◂}$ under volume control. Volume‐controlled measurements of the actuated (blue) membrane used in the parallel setup are included in the bottom graph and are used to estimate the shear modulus of the rubber (G=610 kPa). (B) Schematic of experimental assembly with two identical membranes placed in parallel and mounted face‐to‐face. Each membrane can be independently accessed via external tubing: port (a) connects the actuated (blue) membrane to the external environment, and port (b) does the same for the responsive (orange) membrane. Both membranes are fluidically connected through the cavity filled with an incompressible fluid and accessed through port (c), allowing control of the volume preset $\Delta v^{*}$, which determines the relative horizontal shift between the individual pv‐curves. In the remainder of the paper, port (a) is used to supply the system with a driving pressure $p_{tot}$. (C) Predicted normalized pv‐curves of the total parallel system for various values of the normalized volume preset $\Delta v^{*}/r_{o}^{3}$, showing how the combined system response evolves as a function of the volume preset. (D) Predicted and measured normalized pv‐curves of the total parallel system for two different normalized volume presets: $\Delta v^{*}/r_{o}^{3}=1.2\;\left[-\right]$ (top, rectangle in C) and $\Delta v^{*}/r_{o}^{3}=1.4\;\left[-\right]$ (bottom, circle in C). Predictions are constructed by vertically adding simulated normalized pv‐curves of the two individual membranes after applying the volume preset (horizontal translation). These predictions are validated using volume‐controlled measurements of the full system. Numbered arrows on the top graph indicate snapping events.

The presetting approach and analysis tools developed in this work can be applied to arbitrary connected inflatable structures. However, a particularly interesting case arises when two fluidic hysterons are connected in parallel and preset in volume. We use a truncated conical membrane as an inflatable fluidic hysteron [13], with its geometry detailed in Section S1. Their associated pv‐curve is displayed on Figure 2A and was obtained using the finite element method (ABAQUS/standard), as detailed in [13]. Pressure is normalized by the shear modulus G of the rubber and volume by the cubed radius $r_{o}$ of the membrane. This membrane exhibits hysteresis under both pressure‐ and volume‐controlled loading, as demonstrated on the top and bottom subfigures, respectively. Two such inflatables are mounted face‐to‐face in a rigid housing with incompressible fluid in between (see Figure 2B). Port (a) is used for loading, port (b) is open to the atmosphere, and port (c) is used for volume presetting. The total pv‐curve of the system, observed via port (a), can be obtained using Equation (4), as shown in Figure 2C for different volume presets $\Delta v^{*}$. Two interesting observations can be made: *(i)* The resulting pv‐curves are symmetric around the origin, due to the geometric symmetry of the system; *(ii)* the complexity of the pv‐curve changes with the preset volume, ranging from single uninterrupted paths in pv‐space at low and high preset volumes, to multiple intersecting paths when $2\Delta v_{◂}<\Delta v^{*}<2\Delta v_{▸}$, with $\Delta v_{◂}$ and $\Delta v_{▸}$ being the volume limit points, as depicted on Figure 2A (see also Section S3.2).

To validate these results, we first fabricate two identical truncated conical membranes out of a two‐component addition‐cure silicone rubber (Silicones and More, Shore A 50), and characterize their individual pv‐curves by inflating them with water under volume control using a syringe drive and measuring the resulting pressure. In Figure 2A (bottom), we compare the numerical (red and dark blue curves) and experimental (light blue curve) pv‐curves and see a good agreement between both, with isochoric snapping at the volumetric limit points, indicated by $\Delta v_{◂}$ and $\Delta v_{▸}$. Next, we validate the parallel connection of two such truncated conical membranes by mounting them face‐to‐face in a rigid housing and applying a volume preset using a dedicated syringe drive (see Section S2.1 for details). The global pv‐characteristics of the parallel‐connected membranes, as measured using the same protocol as before, are displayed in Figure 2D for two presetting values, corresponding to $\Delta v^{*}/r_{o}^{3}=1.2\;\left[-\right]$ and $\Delta v^{*}/r_{o}^{3}=1.4\;\left[-\right]$. In simpler cases ($\Delta v^{*}/r_{o}^{3}=1.2\;\left[-\right]$), the experimental curve (in red color) follows the theoretical curve that is based on the parallel coupling of simulated actuators. For example, the first snapping event (1 on Figure 2D‐top) corresponds to the snapping of the orange membrane, as it occurs at its volume limit point. However, for a larger presetting value of $\Delta v^{*}/r_{o}^{3}=1.4\;\left[-\right]$, snapping events are more difficult to trace back to the volume limit points of individual membranes. Further, it is less intuitive to predict which part of the curve the system will snap to, as multiple stable crossing points exist at the same volume. Although the general framework allows for computing the total pv‐curves of parallel and serial inflatable systems, in general, it does not lead to insights into the behavior of the total system during loading and unloading.

### 1.4 Predicting Actuation Sequence of Fluidic Hysterons

To gain insight into the complex dynamics of parallel‐connected fluidic hysterons, we first analyze the behavior of an individual hysteron. In accordance with [27, 35], a hysteron can be modeled as having two states, down (state 0) and up (state 1), with two associated switching fields. In the fluidic case, the hysteron can be actuated either through pressure control or volume control. Under pressure control, the switching fields are the pressure thresholds $p_{▴}$ and $p_{▾}$, which must be exceeded to transition between the two states, as illustrated at the top of Figure 2A. Under volume control, the corresponding switching fields are the volume thresholds $\Delta v_{▸}$ and $\Delta v_{◂}$, shown at the bottom of Figure 2A.

**FIGURE 3:**
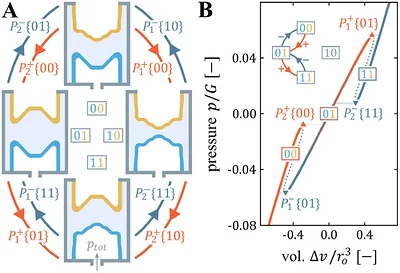
State Transitions of Parallel Fluidic Hysterons (A) Full state diagram of the parallel system, illustrating the four collective states S={00},{01},{10}, and {11} for the two parallel hysterons. The eight arrows represent transitions between these states, each associated with a switching pressure $P_{i}^{\pm }\left\{S\right\}$ under a pressure driving field $p_{tot}$. These switching pressures define the values of $p_{tot}$ required to induce a transition between collective states. (B) Identification of switching pressures from the predicted normalized pv‐curve of the parallel system for a normalized volume preset of $\Delta v^{*}/r_{o}^{3}=0.7\;\left[-\right]$. The inset shows the corresponding state diagram. For this specific preset, state {10} is unstable.

In the parallel configuration, the collective state of the two hysterons can be denoted as $S=\{s_{1},s_{2}\}$, where $s_{1}$ refers to the state of the actuated (blue) membrane and $s_{2}$ to the responsive (orange) membrane. The four possible collective states, as shown in Figure 3A, are {00}, {01}, {10} and {11}. When driving the combined system with a pressure $p_{tot}$, transitions between collective states occur at discrete switching pressures, denoted as $P_{i}^{\pm }\left\{S\right\}$. The subscript i indicates which hysteron is switching: i=1 for the actuated (blue) membrane, and i=2 for the responsive (orange) one. The superscript ± denotes the direction of the transition: ‘+’ for transitioning from state 0 to state 1, ‘−’ for the ‘1 to 0’‐transition. S shows the collective state from which the transition occurs. For two hysterons, this results in eight possible switching pressures, as shown in Figure 3A. The arrows indicate the relationship between each switching pressure and its corresponding state change. For a simpler parallel connection, the state diagrams and switching pressures can be directly deduced from the system's combined pv‐curve. As an example, we refer to Figure 3B where the pv‐curve is displayed for a parallel system with a preset volume of $\Delta v^{*}/r_{o}^{3}=0.7\;\left[-\right]$. At very low pressures, both membranes are down, being in the {00} state. Upon inflation, the system snaps to state {01} at pressure $P_{2}^{+}\left\{00\right\}$ and snaps to state {11} at pressure $P_{1}^{+}\left\{01\right\}$. During subsequent deflation, the system snaps back to state {01} at pressure $P_{2}^{-}\left\{11\right\}$ and to state {00} at pressure $P_{1}^{-}\left\{01\right\}$. State {10} is not reached, indicating its instability under these preset condition. The resulting system state diagram is shown as an inset in Figure 3B, where a red arrow indicates inflation, and a blue arrow indicates deflation.

**FIGURE 4:**
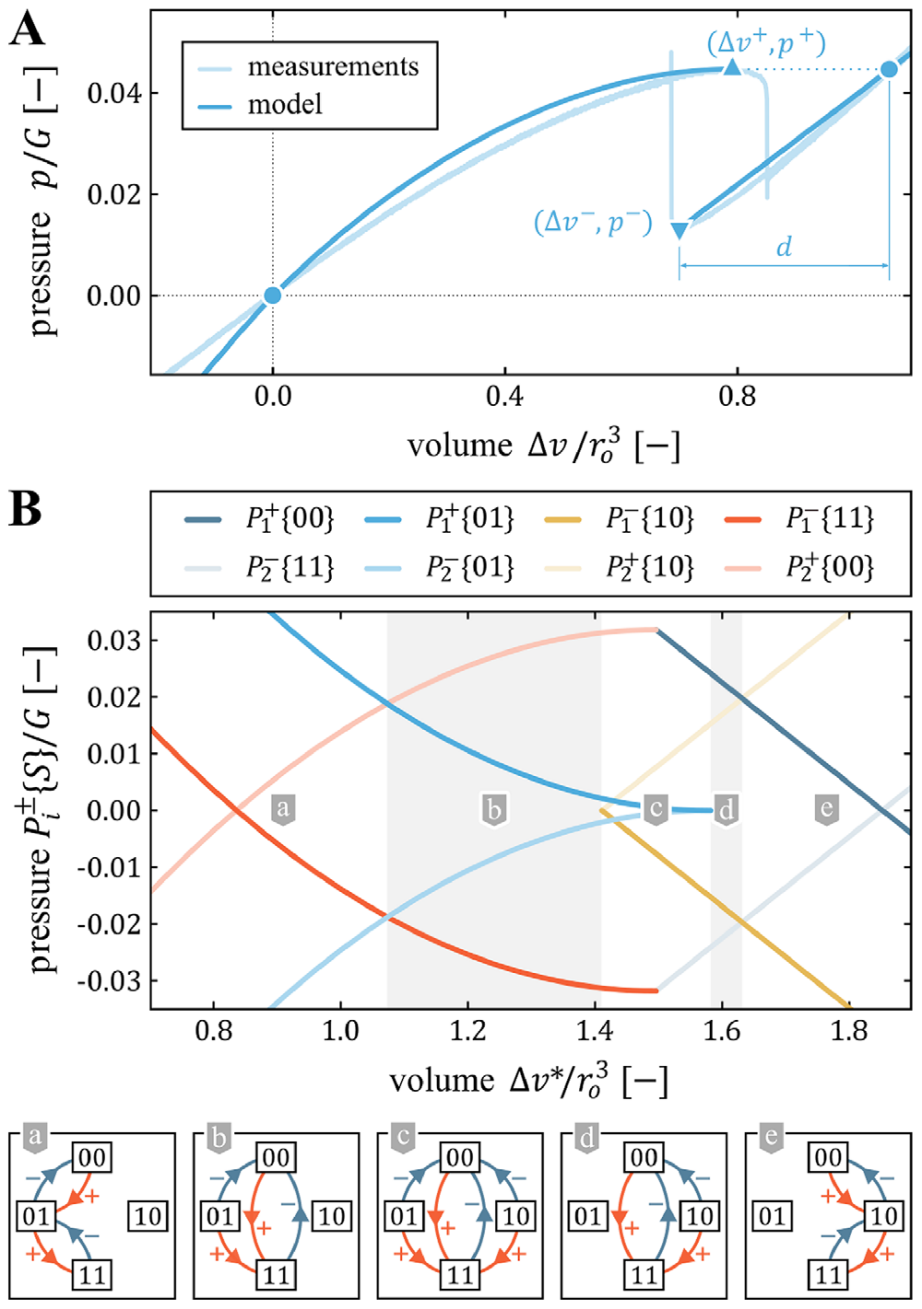
Analytical Model of Parallel Fluidic Hysterons. (A) Proposed parabolic‐linear model fitted to experimental normalized pv‐curve of the actuated (blue) membrane. The markers indicate the specific data points used for model fitting. This model captures the nonlinear characteristics required to predict state transitions analytically in the parallel setup. (B) Graphical representation of the resulting normalized analytical switching pressures $P_{i}^{\pm }\left\{S\right\}/G$ as a function of the normalized volume preset $\Delta v^{*}/r_{o}^{3}$ (top) (Equations 8 and 9), with five corresponding state diagrams (bottom). Each state diagram (a)–(e) reflects the stability and accessibility of the four possible states for a given range of $\Delta v^{*}$. Gray regions link each state diagram to the corresponding volume presets, highlighting how the system's switching logic evolves with the preset.

For more intricate systems, determining the system's state diagram solely using the pv‐curve becomes less straightforward. To address this, we introduce a reduced‐order model where the pv‐curve of each individual membrane is approximated using a piecewise polynomial model. Figure 4A shows the measured pv‐curve of the actuated (blue) membrane alongside the fitted model, which incorporates both a parabolic and a linear segment. In contrast to Liu et al. [27], where a linear‐linear approximation was used, the parabolic‐linear approximation significantly improves modeling accuracy (see Section S3.4). For the blue membrane, the parabolic function describes the zero state $s_{1}=0$ and is defined by the origin (0,0) and the snap‐through point ($\Delta v^{+},p^{+}$), which corresponds to the parabola's apex. The pressure threshold satisfies $p^{+}=p_{▴}$, since the system is actuated using pressure control. The linear function describes the one state $s_{1}=1$ and interpolates between the snap‐back point ($\Delta v^{-},p^{-}$), where $p^{-}=p_{▾}$, and the point to which snap‐through transitions ($\Delta v^{-}+d,p^{+}$). As a result, five parameters are needed to fit the parabolic‐linear approximation: $\Delta v^{+}$, $p^{+}$, $\Delta v^{-}$, $p^{-}$, and d (see Figure 4A). The corresponding model equations for the blue membrane are:

(5) $$\begin{array}{cc} \text{for}\;s_{1}=0: & \;\;\;p_{1}=-p^{+}\left[{\left(\frac{\Delta v_{1}}{\Delta v^{+}}\right)}^{2}-2\frac{\Delta v_{1}}{\Delta v^{+}}\right]\\ \text{for}\;s_{1}=1: & \;\;\;p_{1}=\frac{p^{+}-p^{-}}{d}\left(\Delta v_{1}-\Delta v^{-}\right)+p^{-} \end{array}$$

For the inverted, orange membrane, the parabolic function describes $s_{2}=1$, and the linear function describes $s_{2}=0$. Additionally, the pressure–volume curve is mirrored around the origin to reflect the opposing orientation of the membranes:

(6) $$\begin{array}{cc} \text{for}\;s_{2}=0: & \;\;\;p_{2}=\frac{p^{+}-p^{-}}{d}\left(\Delta v_{2}+\Delta v^{-}\right)-p^{-}\\ \text{for}\;s_{2}=1: & \;\;\;p_{2}=p^{+}\left[{\left(\frac{\Delta v_{2}}{\Delta v^{+}}\right)}^{2}-2\frac{\Delta v_{2}}{\Delta v^{+}}\right] \end{array}$$

With the presented model, analytical expressions for the switching pressures $P_{i}^{\pm }\left\{S\right\}$ can be derived following the approach of Liu et al. [27]. The pressure $P_{i}^{\pm }\left\{S\right\}$ is the pressure at which the *i*th hysteron reaches its switching threshold $p_{i}^{\pm }$. Using the parallel theory of Equation (4) and the model expressions defined in Equations (5) and (6), the following relations can be stated:

(7) $$P_{i}^{\pm }\left\{S\right\}=P_{i}\left\{S\right\}\left(p_{i}=p_{i}^{\pm },\Delta v^{*}\right)$$

Thus, for the first hysteron, these analytical expressions become:

(8) $$\begin{array}{cc} P_{1}^{+}\left\{00\right\}= & \;\left(p^{+}-p^{-}\right)\left[1+\frac{\Delta v^{+}+\Delta v^{-}-\Delta v^{*}}{d}\right]\\ P_{1}^{+}\left\{01\right\}= & \;p^{+}{\left[2-\frac{\Delta v^{*}}{\Delta v^{+}}\right]}^{2}\\ P_{1}^{-}\left\{10\right\}= & \;\frac{p^{+}-p^{-}}{d}\left(2\Delta v^{-}-\Delta v^{*}\right)\\ P_{1}^{-}\left\{11\right\}= & \;p^{-}-p^{+}+p^{+}{\left[1+\frac{\Delta v^{-}-\Delta v^{*}}{\Delta v^{+}}\right]}^{2} \end{array}$$

Due to the symmetry of the system, the switching pressures of the second hysteron ($P_{2}^{\pm }\left\{S\right\}$) can be related to those of the first one:

(9) $$\begin{array}{cc} P_{2}^{+}\left\{00\right\}= & \;-P_{1}^{-}\left\{11\right\}\\ P_{2}^{+}\left\{10\right\}= & \;-P_{1}^{-}\left\{10\right\}\\ P_{2}^{-}\left\{01\right\}= & \;-P_{1}^{+}\left\{01\right\}\\ P_{2}^{-}\left\{11\right\}= & \;-P_{1}^{+}\left\{00\right\} \end{array}$$

The details of the derivation of these analytical expressions can be found in Section S3.3, where the switching pressures are first derived for a general asymmetric two‐hysteron system, after which symmetry assumptions are imposed to obtain the expressions considered here in the main text. The switching pressures are plotted on Figure 4B as a function of the preset volume $\Delta v^{*}$ for a parallel system of truncated conical membranes with $p^{+}$, $\Delta v^{+}$, $p^{-}$, $\Delta v^{-}$, and d derived from the experimental pv‐curves of the blue and orange membranes. The symmetry of the system (see Equation 9) is also reflected in the switching field graph (Figure 4B).

The ordering of the switching pressures directly determines the sequence of state transitions and thus the topology of the resulting state diagram. These state diagrams are constructed by analyzing the stability ranges of each state as a function of the preset volume $\Delta v^{*}$, as detailed in Section S3.5. This analysis reveals that varying this preset volume gives rise to five distinct state diagrams, labeled (a) through (e). The zones corresponding to different state diagrams are indicated by gray shading in Figure 4B, with corresponding state diagrams plotted below the graph.

The locations of the zone boundaries can be determined analytically by evaluating when specific switching pressures coincide or change sign. Using the expressions for the switching pressures given in Equations (8) and (9), these boundaries are derived in Section S3.6 and can be summarized as:

(10) $$\begin{array}{cc} (a)-(b): & \;\;\;\Delta v^{*}=\frac{\Delta v^{-}+3\Delta v^{+}}{2}\\ & -\frac{1}{2}\sqrt{-{\left(\Delta v^{-}\right)}^{2}+2\Delta v^{-}\Delta v^{+}+\left(1-2\frac{p^{-}}{p^{+}}\right){\left(\Delta v^{+}\right)}^{2}}\\ (b)-(c): & \;\;\;\Delta v^{*}=2\Delta v^{-}\\ (c)-(d): & \;\;\;\Delta v^{*}=2\Delta v^{+}\\ (d)-(e): & \;\;\;\Delta v^{*}=\frac{3\Delta v^{-}+\Delta v^{+}+d}{2} \end{array}$$

At low preset volumes ($\Delta v^{*}/r_{o}^{3}<1.08\;\left[-\right]$, zone (a)), the switching pressure $P_{2}^{+}\left\{00\right\}$ has a smaller magnitude than $P_{1}^{+}\left\{01\right\}$, rendering state S={10} unstable. Thus, upon inflation S={01} is reached from S={00}. Further inflation will result in a transition to S={11}. Deflation follows the reverse path, again passing through S={01}. At high preset volumes ($\Delta v^{*}/r_{o}^{3}>1.63\;\left[-\right]$, zone (e)) the system behaves similarly, transitioning via S={10} instead of state S={01}. In both outer zones, switching occurs sequentially without collective effects. For intermediate preset volumes ($1.08\;\left[-\right]<\Delta v^{*}/r_{o}^{3}<1.63\;\left[-\right]$, zones (b) through (d)), *avalanches* are present, where the switching of one of the membranes triggers the immediate transition of the other. For example, in zone (b), $P_{2}^{+}\left\{00\right\}$ has a higher magnitude than $P_{1}^{+}\left\{01\right\}$, meaning that after switching from S={00} to S={10}, the system instantaneously transitions to S={11} because $P_{1}^{+}\left\{01\right\}$ has already been exceeded. These avalanches give rise to collective states that are stable yet not reachable from the saturated states {00} and {11}. Specifically, state {01} in zones (b) and (c), and state {10} in zones (c) and (d) can be considered *Garden‐of‐Eden* states [35] that once exited, cannot be reached again.

Equation (10) can thus be used to determine the state diagram zone boundaries for a given fluidic hysteron. Inversely, these analytical expressions provide a design tool to tailor the global state space behavior by means of tuning the hysteron parameters. As such, one can selectively enlarge or suppress specific zones and thereby control which state‐diagram topology is realized. A detailed analysis of how the zone boundaries depend on the hysteron parameters is provided in Section S3.6, with the following general conclusion: (i) Zones (a) and (e) are unbounded toward small and large preset volumes, respectively; that is, for sufficiently small or large values of the preset volume $\Delta v^{*}$, the system necessarily falls into zone (a) or zone (e). (ii) For inflatable hysterons with similar characteristics as studied here, zone (b) generally spans a broad interval of $\Delta v^{*}$, while the intermediate zones (c) and (d) occupy comparatively narrow ranges. (iii) Increasing the difference $\Delta v^{+}-\Delta v^{-}$ enlarges zone (c) but simultaneously reduces the extent of zone (d). As a result, zone (d) may disappear entirely for sufficiently large $\Delta v^{+}$ or small $\Delta v^{-}$. (iv) Sufficiently small values of $\Delta v^{-}$ can eliminate zone (b). (v) For hysterons with $\Delta v^{+}-\Delta v^{-}<0$ (i.e., the snapping‐up volume is smaller than the snapping‐down volume), zone (c) does not exist.

We also refer the reader to Section S3.3, where a visual comparison of the switching pressures and state diagrams is presented for the symmetric and asymmetric two‐hysteron cases. This comparison shows that asymmetry introduces additional state‐diagram topologies that are absent in the symmetric configuration.

Overall, the proposed approach provides insight into the state‐space behavior of parallel (hysteron) systems, without the need to compute or interpret the full pv‐curve. In addition, we show that the volume presetting ($\Delta v^{*}$) is a powerful tuning parameter that can be used to select the active state‐diagram topology of the system prior to actual operation.

### 1.5 Experimental Validation

**FIGURE 5:**
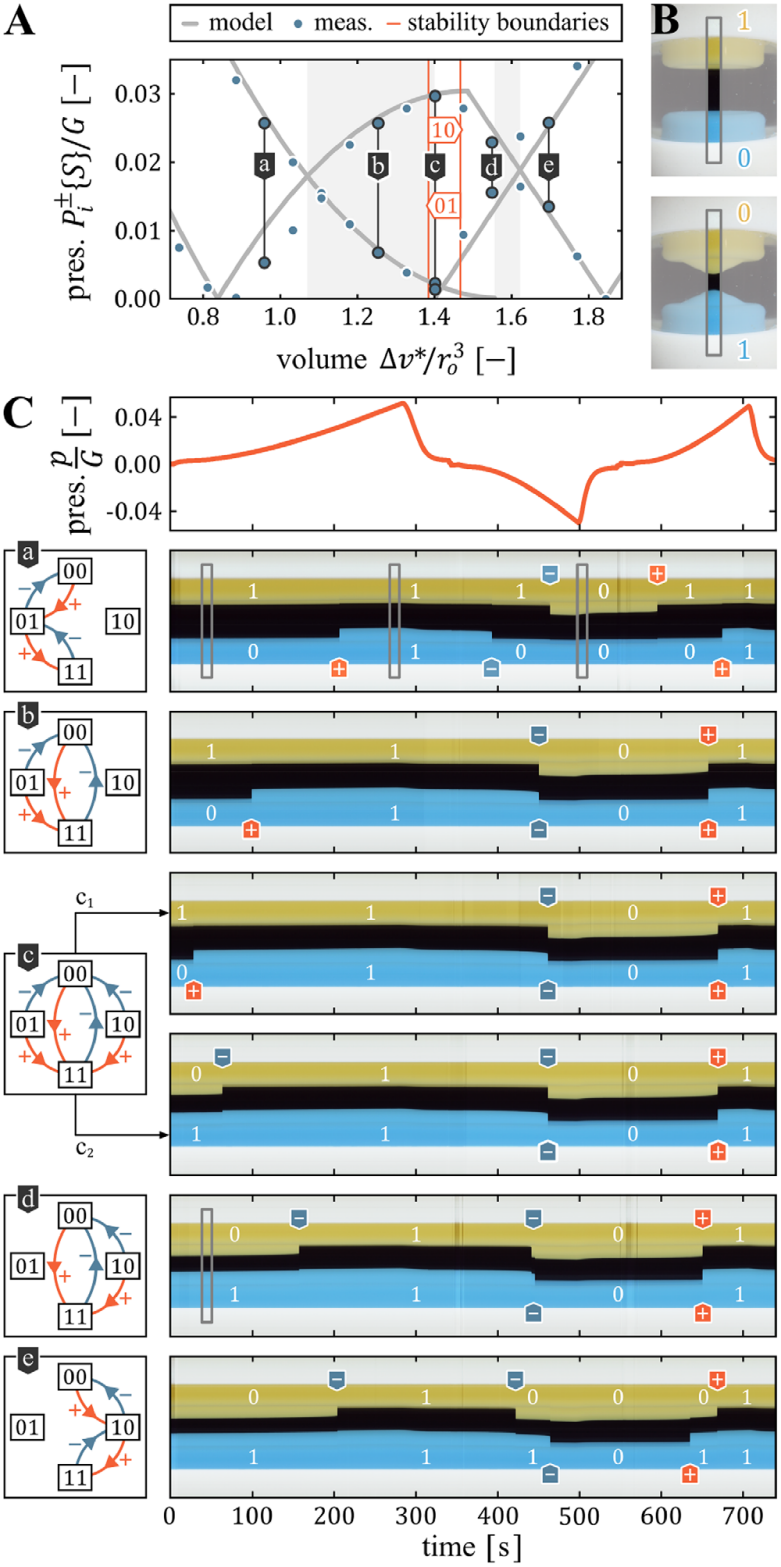
Experimental Validation of the Fluidic Hysteron Model. (A) Measured normalized switching pressures $P_{i}^{\pm }\left\{S\right\}/G$ of the parallel system as a function of the normalized volume preset $\Delta v^{*}/r_{o}^{3}$, validating the analytical expressions in Equations (8) and (9), as introduced in Figure 4B. Only the positive switching transitions are shown; negative switching pressures can be obtained by taking the mirror image. Blue circles represent pressure‐controlled tests using air. Stability boundaries of the intermediate states {10} and {01} are determined from volume‐controlled tests. Measurement details are given in Sections S2.2 and S2.3. Five data sets (a)–(e), each corresponding to a different state diagram, are selected for further analysis. (B) Snapshots of the collective states {01} and {10} of the parallel system, captured during actuation (see Figure S2 for {00} and {11}). The central pixel line of each video frame (highlighted in the snapshots) is extracted and used in panel C. (C) The system is actuated at five preset volumes $\Delta v^{*}$, each corresponding to a different state diagram. A predetermined pressure input (top panel) is applied, and videos are recorded during actuation. For each video, the extracted pixel lines are placed sequentially to create photo‐finish‐like images, revealing the system's temporal evolution. Collective states and transitions are identified from these images. Gray boxes correspond to the snapshots shown in B and Figure S2. For state diagram (c), both {10} and {01} are stable for $p_{tot}=0$, allowing two separate experiments: ($c_{1}$) starts from state {01}, while ($c_{2}$) starts from state {10}; both subjected to the same pressure profile.

To experimentally validate the proposed theory, we employ the parallel membrane system shown in Figure 2B. The preset volume is controlled using a syringe driven by a micrometer screw. The loading pressure, which is applied to the blue membrane, is controlled using a proportional pressure regulator (VEAB, Festo) and measured by a pressure sensor (A‐10, Wika). The experiment consists of applying a specified pressure profile and recording the pressures at which the membranes switch between states (the switching pressures $P_{i}^{\pm }\left\{S\right\}$). These measurements allow identification of which state diagrams occur at specific volume presets and serve as a basis for comparison with the analytically predicted zones. The results are shown in Figure 5A, where experimental data are plotted alongside the model predictions for positive switching pressures. Negative switching pressures can be obtained by taking the mirror image due to symmetry. The measured switching pressures (blue dots) are obtained from pressure‐controlled tests using air (see Section S2.2). The stability boundaries (orange lines) are determined using a volume‐controlled test using water (see Section S2.3) and indicate the volume presetting thresholds beyond which the asymmetric states {10} and {01} become unstable. Specifically, below a volume difference of $\Delta v^{*}/r_{o}^{3}=1.41\;\left[-\right]$, the {10} state is unstable, whereas above $\Delta v^{*}/r_{o}^{3}=1.51\;\left[-\right]$, the {01} state becomes unstable. From these measurements, it can be concluded that *(i)* the experimental switching pressures show good agreement with the modeled values; *(ii)* the transition between zones (b) and (c), which coincides with the stability boundary of state {10}, is accurately captured; *(iii)* experimentally, the {01} state becomes unstable at a smaller volume preset than predicted by the analytical model, resulting in a leftward shift of the experimentally observed (c)–(d) boundary relative to the prediction. Based on the analytical sensitivity analysis (Section S3.6), it can be concluded that the (c)‐(d) boundary depends only on $\Delta v^{+}$, which is challenging to accurately determine experimentally. Overall, the transitions between state diagrams are well captured by the model, demonstrating its effectiveness in predicting the overall behavior of the system.

To further show the system's capabilities and illustrate how varying preset volumes result in substantially different actuation sequences, the setup is operated under a prescribed pressure profile (Figure 5C) with different volume presets applied. Five preset volumes are applied, each belonging to a different state diagram zone, as indicated in Figure 5A by black vertical lines. To visualize the behavior of the system, we capture the deformations of both membranes using strip photography, where a single vertical pixel line is extracted (see as illustrated in Figure 5B and Figure S2) and chronologically concatenated. This method allows for tracking of membrane positions over time and clear identification of switching events. Further details of the experimental procedures and data processing are provided in Sections S2.2 and S2.3. The resulting images (Figure 5C) show that in zones (a) through (c), state {01} is stable. In zones (c) through (e), state {10} is stable. For zone (c), both state {01} and state {10} can be stable at the beginning of the pressure profile, and external manipulation was used to bring the system into these Garden‐of‐Eden states. Zones (b) through (d) are characterized by avalanches where state {00} directly leads to state {11} upon inflation and vice versad during deflation. In general the preset volume can be used to transition from a state diagram where state {01} is both stable and accessible while state {10} is not stable (low preset volumes) to a state diagram where state {10} is both stable and accessible while state {10} is not stable (high preset volumes). Increasing the preset volume causes state {01} to lose stability via a Garden‐of‐Eden state, while state {10} will gain stability, again via a Garden‐of‐Eden state.

### 1.6 Demonstrator

**FIGURE 6:**
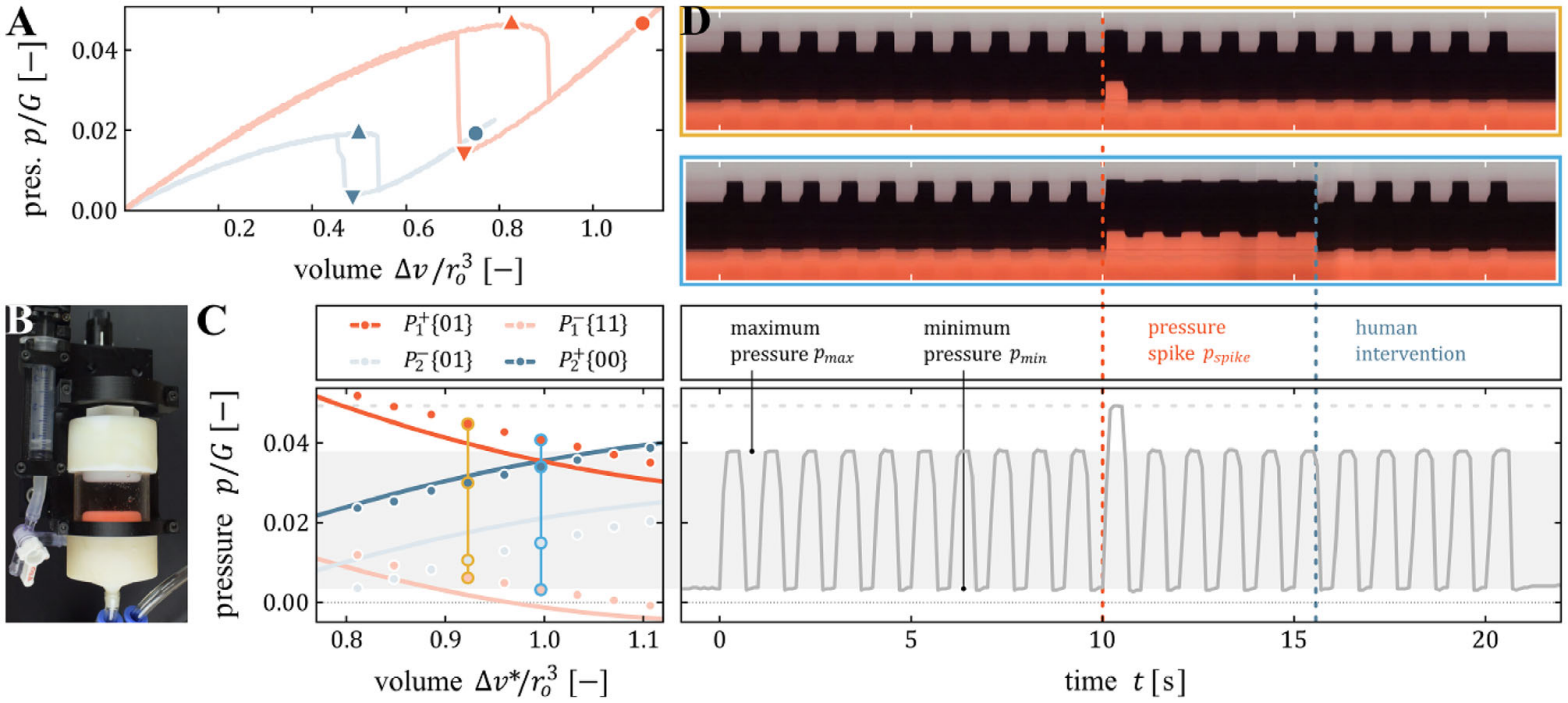
Surge Protector Demonstrator. (A) Volume‐controlled measured pressure–volume (pv) curves of the two fluidic hysterons used in the demonstrator: the right (red) membrane and the left (blue) membrane. For each hysteron, the upward switching point ($\Delta v^{+}$, $p^{+}$) is indicated by an upward triangle ▴, the downward switching point ($\Delta v^{-}$, $p^{-}$) by a downward arrow ▾, and the snap‐through point ($\Delta v^{-}+d$, $p^{+}$) by a circle •. (B) Experimental setup of the parallel‐connected membrane system. The bottom (red) membrane is actuated using pressure control, while the top (blue) membrane is open to the atmosphere. The membranes are coupled through an incompressible fluid (water), and the preset volume $\Delta v^{*}$ is adjusted using the syringe on the left. (C) Left graph: Predicted (lines) and experimentally measured (circles) switching pressures of the asymmetric parallel two‐hysteron system as a function of preset volume $\Delta v^{*}$, obtained from volume‐controlled measurements using water. Two preset volumes are highlighted: $\Delta v_{1}^{*}/r_{o}^{3}=0.922\;\left[-\right]$ (orange) and $\Delta v_{2}^{*}/r_{o}^{3}=0.996\;\left[-\right]$ (blue), corresponding to the two cases shown in Video S1. Right graph: Applied pressure input over time, consisting of oscillatory loading between $p_{min}$ and $p_{max}$ at 1 Hz, a pressure spike $p_{spike}$ at t=10 s, and a manual intervention at t=15.5 s for the second preset. (D) Time‐resolved photo‐finish‐like images of the membrane response synchronized with the pressure input shown in panel C. The top row corresponds to $\Delta v_{1}^{*}$ and shows recovery of oscillatory behavior after the pressure spike. The bottom row corresponds to $\Delta v_{2}^{*}$, where the same spike traps the system in the {11} state, suppressing oscillations until a brief manual perturbation resets the system. Together, the figure demonstrates how the preset volume selects distinct state‐diagram regimes and enables surge‐protection‐like behavior without modifying the pressure input or system connectivity.

To demonstrate a functional use case enabled by interacting parallel hysterons, we introduce a pressure surge‐protector demonstrator. In contrast to the symmetric configuration, the two identical membranes are replaced by two deliberately different fluidic hysterons (shown in Figure 6B), resulting in an asymmetric parallel system characterized by ten hysteron parameters ($p_{1}^{+}$, $\Delta v_{1}^{+}$, $p_{1}^{-}$, $\Delta v_{1}^{-}$, $d_{1}$, $p_{2}^{+}$, $\Delta v_{2}^{+}$, $p_{2}^{-}$, $\Delta v_{2}^{-}$, and $d_{2}$).

The pressure–volume curves of both hysterons are measured and analyzed following the same procedure used for the symmetric system (Figure 4A), allowing all parameters to be extracted experimentally, as shown in Figure 6A. These parameters are then incorporated into the analytical framework to compute the switching pressures $P_{i}^{\pm }\left\{S\right\}$ of the asymmetric parallel system as functions of the preset volume $\Delta v^{*}$. The resulting analytical predictions are shown in the left graph of Figure 6C, together with volume‐controlled experimental measurements of the switching pressures. Based on these analytical predictions, two preset volumes are selected: $\Delta v_{1}^{*}/r_{o}^{3}=0.922\;\left[-\right]$ and $\Delta v_{2}^{*}/r_{o}^{3}=0.996\;\left[-\right]$ highlighted in orange and blue, respectively, in Figure 6C. These presets can be switched manually using the adjustment knob located on top of the experimental setup (Figure 6B).

For both preset volumes, the system is subjected to the same pressure input, consisting of oscillations between $p_{min}$ and $p_{max}$ at 1 Hz, as shown in the right graph of Figure 6C. The oscillatory pressure bounds are chosen such that

(11) $$\begin{array}{c} P_{2}^{+}\left\{00\right\}\left(\Delta v_{2}^{*}\right)<p_{max}<P_{1}^{+}\left\{01\right\}\left(\Delta v_{2}^{*}\right)\\ P_{1}^{-}\left\{11\right\}\left(\Delta v_{2}^{*}\right)<p_{min}<P_{2}^{-}\left\{01\right\}\left(\Delta v_{1}^{*}\right) \end{array}$$

Under these conditions, the membrane connected to the atmosphere (blue) undergoes periodic snapping during normal operation for both preset volumes, corresponding to oscillations of the collective system between states {00} and {01}. At t=10 s, a pressure spike $p_{spike}$ is applied, causing the actuated (red) hysteron to snap upward in both preset configurations and temporarily driving the system into state {11}. When the pressure returns to the oscillatory regime, the subsequent system response depends on the preset volume. For the system at preset volume $\Delta v_{1}^{*}$, the red hysteron snaps back down, allowing the blue hysteron to resume oscillatory switching. In contrast, for the system at preset volume $\Delta v_{2}^{*}$, the red hysteron remains latched in the upward state after the spike, suppressing further oscillations and effectively isolating the system from the oscillatory input. At t=15.5 s, a brief mechanical perturbation is manually applied to reset both membranes to the {00} state, after which oscillatory behavior resumes. A video of this demonstrator is provided as Video S1.

This demonstrator illustrates how preset volume selection can determine whether pressure surges are absorbed and forgotten or permanently latch the system into a protective state. Importantly, this functionality emerges without altering the pressure input, control architecture, or system connectivity, highlighting the practical utility of volume‐constrained parallel coupling for programmable, memory‐enabled soft systems. Notably, this surge‐protection behavior relies on sensitivity to transient pressure excursions, a feature intrinsic to volume‐constrained parallel coupling and difficult to reproduce in pressure‐shared (series) inflatable architectures, which primarily respond to cumulative volume changes rather than instantaneous pressure spikes.

## 2 Conclusion

To encode functionality in pneumatic networks, a clear understanding of how inflatable structures interact is essential. In this work, we present a theoretical and experimental framework for understanding and predicting the behavior of connected inflatables. Starting from basic serial and parallel connections, we introduce the concept of presetting internal volumes and pressures to modulate system response. This framework is applied to parallel‐connected fluidic hysterons, which are hysteretic elements with nonlinear behavior that can serve as memory components in pneumatic networks. By presetting internal volumes, the overall response of the system can be altered. To facilitate predictive modeling, a simplified parabolic‐linear representation of the fluidic hysteron is employed. Experimental validation shows strong agreement between the model's predictions and the observed system behavior, confirming the validity of the approach. Importantly, this approach enables real‐time control of actuation sequences without modifying the physical architecture, connectivity, or loading conditions. Together, these results establish a foundation for designing programmable soft systems with tunable, memory‐rich responses governed by material and structural properties.

## Author Contributions

K.S. and B.G. brainstormed and developed the research idea. K.S. developed the theoretical framework and analytical model, designed and built the experimental setup, and conducted all experiments. K.S. analyzed the data, prepared the figures, and wrote the manuscript. FN.PB. and B.G. contributed to writing and provided feedback on the manuscript. B.G. supervised the research.

## Conflicts of Interest

The authors declare no conflicts of interest.

## Supporting information


**Supporting File 1**: advs76158‐sup‐0001‐SuppMat.pdf.

**Supporting File 2**: advs76158‐sup‐0002‐MovieS1.mp4.

## Data Availability

The data that support the findings of this study are available from the corresponding author upon reasonable request.
