# Supplementary material for: Interacting Parallel Fluidic Hysterons
Source: Adv Sci (Weinh). 2026 Jun 25;13(51):e76158. doi: 10.1002/advs.76158 (PMC13336442; doi:10.1002/advs.76158)
Supplement: Supplementary file 1 [file ADVS-13-e76158-s001.pdf]

Supplementary Materials

Interacting parallel fluidic hysterons

Katrien Stinissen, Franco Nicolas Piñan Basualdo, Benjamin Gorissen

E-mail: benjamin.gorissen@kuleuven.be

This PDF file includes:

Supplementary text
Figs. S1 to S10
Caption for Video S1
References for SI reference citations

Other supplementary materials for this manuscript include the following:

Video S1

Supporting Information Text

S1. Fabrication Parallel Membranes

The two truncated conical membranes were fabricated using an addition-curing silicone rubber (Shore A 50, Silicones And More) and cast in custom brass molds previously developed by our group. Detailed dimensions and manufacturing procedures are provided in the Supporting Information of Van Raemdonck et al. (1). The membrane geometry used in this work corresponds to the design referred to as "disk spring C" in the referenced paper.

The *pv*-curves of the membranes were characterized using a volume-controlled setup, as described in the same reference. From these measurements, the bulk modulus of the membrane material was estimated to be approximately 610 kPa. This value, together with a membrane base radius of $r_o = 10$ mm, was used to non-dimensionalize the *pv*-curves for comparison with simulation results and are shown at the bottom of Figure 2**A**.

The membranes are secured using custom 3D-printed clamps, manufactured on a Stratasys Objet30 Prime 3D printer using an acrylic resin (VERO white plus RGD 835). A transparent PMMA tube encloses the system to enable visual inspection of the water-filled cavity between the membranes. The 3D-printed parts and PMMA-tube are connected using a two-component methacrylate adhesive (MA 300, Plexus). A fluid channel is integrated into the 3D-printed components to allow for precise removal of volume from the cavity. The complete setup is shown in Figure S1.

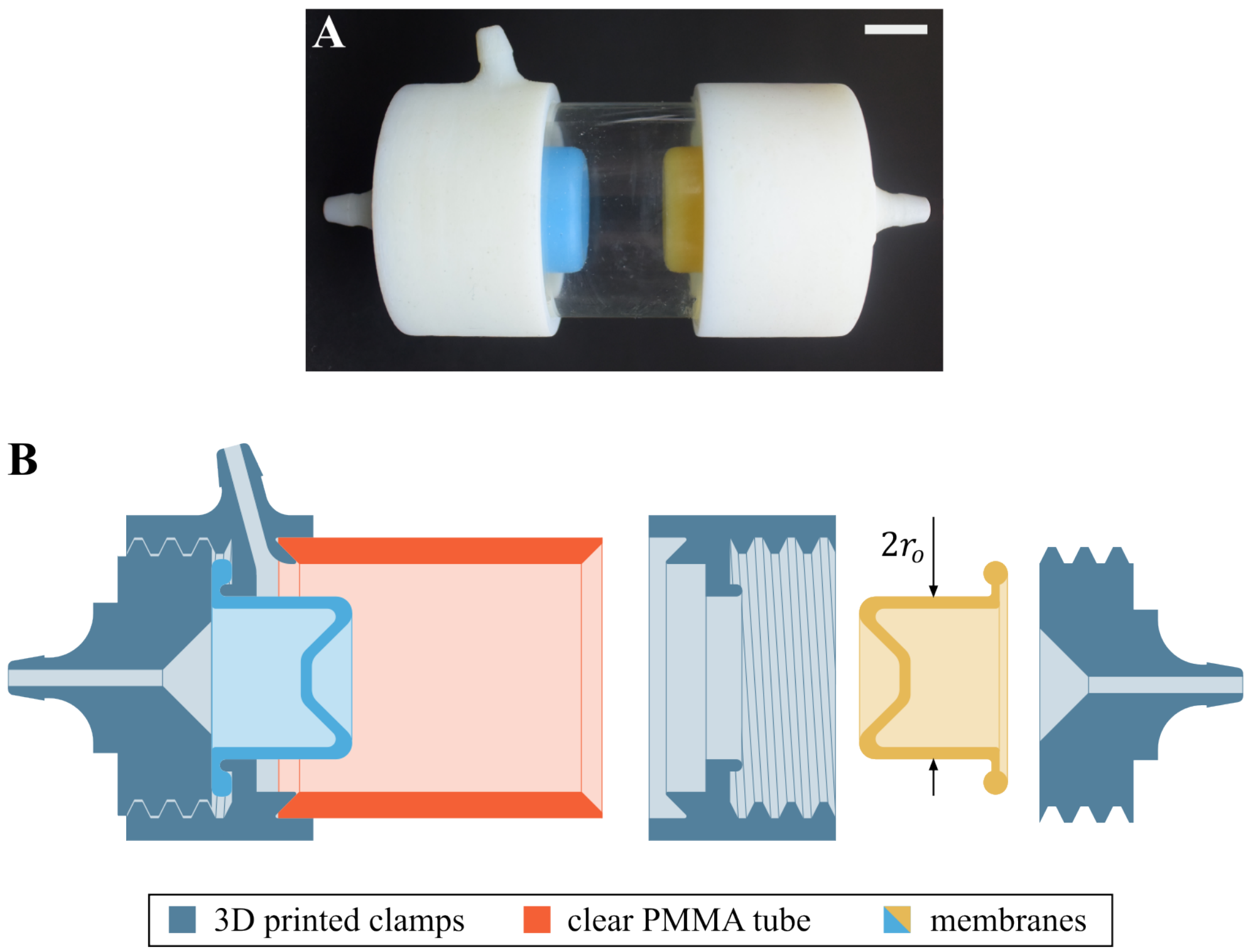

Fig. S1. Parallel Membrane Assembly Parallel assembly used in all experimental validations. The setup consists of two truncated conical rubber membranes, two 3D-printed clamps, and a transparent PMMA tube that connects the membranes in parallel. **A)** Photograph of the experimental assembly. Scale-bar: 10 mm. **B)** Cross-sectional schematic of the experimental assembly. The left side shows the assembled configuration, including the port that provides access to the cavity between the membranes, while the right side presents an exploded view illustrating the individual components: the PMMA-tube, membrane ($r_o = 10$ mm), and 3D-printed parts.

S2. Experiments

S2.1. Parallel Inflatable Structures.

To validate the proposed theory on parallel inflatable structures, volume-controlled experiments were performed using the assembly shown in Figure 2**B**. The cavity between the membranes was initially filled with water. Subsequently, a predefined volume Δv^* was extracted from the cavity via port (c) using a syringe (with $\Delta v^*/r_o^3 = 1.2$ $[-]$ and 1.4 $[-]$ for the top and bottom measurements shown in Figure 2**D**, respectively). After presetting the volume, port (a) was used to perform the *pv*-measurements, following the procedure described in the Supporting Information of Van Raemdonck et al. (1). The acquired data were post-processed to remove the initial experimental drift caused by compliance in the measurement setup, which produces an artificial pressure plateau before the syringe begins to displace fluid.

S2.2. Switching Pressures Fluidic Hysterons.

To measure the switching pressures $P_i^{\pm}\{S\}$ of the parallel fluidic hysterons, pressure-controlled experiments were conducted using air and the assembly shown in Figure 2**B**. Similar to the volume-controlled measurements, the desired preset volume Δv^* was set by extracting water from the cavity using a syringe prior to actuation.

A pressure signal (top panel of Figure 5**C**) was applied to port (a) using a proportional pressure regulator (VEAB-L-26-D2-Q4-V1-1R139 8046305, Festo). A pressure sensor (A-10, Wika) was used to monitor the pressure. A fluidic resistor was placed between the pressure regulator and the pressure sensor, creating a pressure drop during snapping events. This drop enables identification of the switching pressures.

The experiment was repeated and recorded for multiple volume presets, using the same pressure input signal. The resulting switching pressures are plotted as blue circles in Figure 5**A**. For five different volume presets—each corresponding to a distinct zone in Figures 4**B** and 5**A**—the video data were post-processed and are shown in Figure 5**C**. The center pixel row of each video frame was extracted (see Figure S2), and arranged sequentially to generate a photo-finish-like image that visually captures the snapping sequence over time.

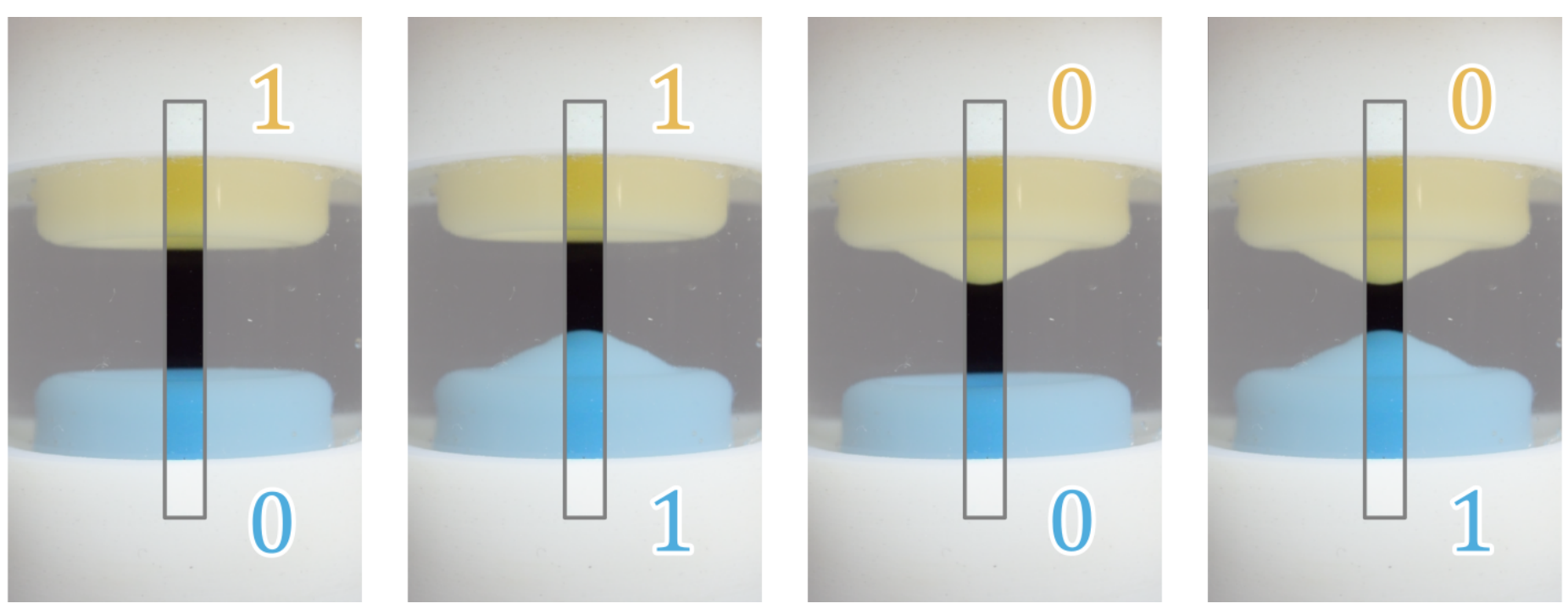

Fig. S2. Snapshots from Pressure-Controlled Experiments Snapshots of the collective states $\{01\}$, $\{11\}$, $\{00\}$ and $\{10\}$ of the parallel system during actuation. The middle pixel line of each video frame (highlighted in the snapshots) is extracted and used in Figure 5**C**.

S2.3. Stability Boundaries.

To determine the stability range of the collective states $S = \{01\}$ and $\{10\}$ when the driving pressure is atmospheric ($p_{tot} = 0$), volume-controlled measurements were performed using water. Volume was extracted through port (c) of the parallel membrane assembly shown in Figure 2**B**, following the procedure described in the Supporting Information of Van Raemdonck et al. (1). The resulting *pv*-data is presented in Figure S3, with the corresponding collective states indicated on the graph. This allows identification of the volume presets $\Delta v^*/r_o^3$ for which each state is stable.

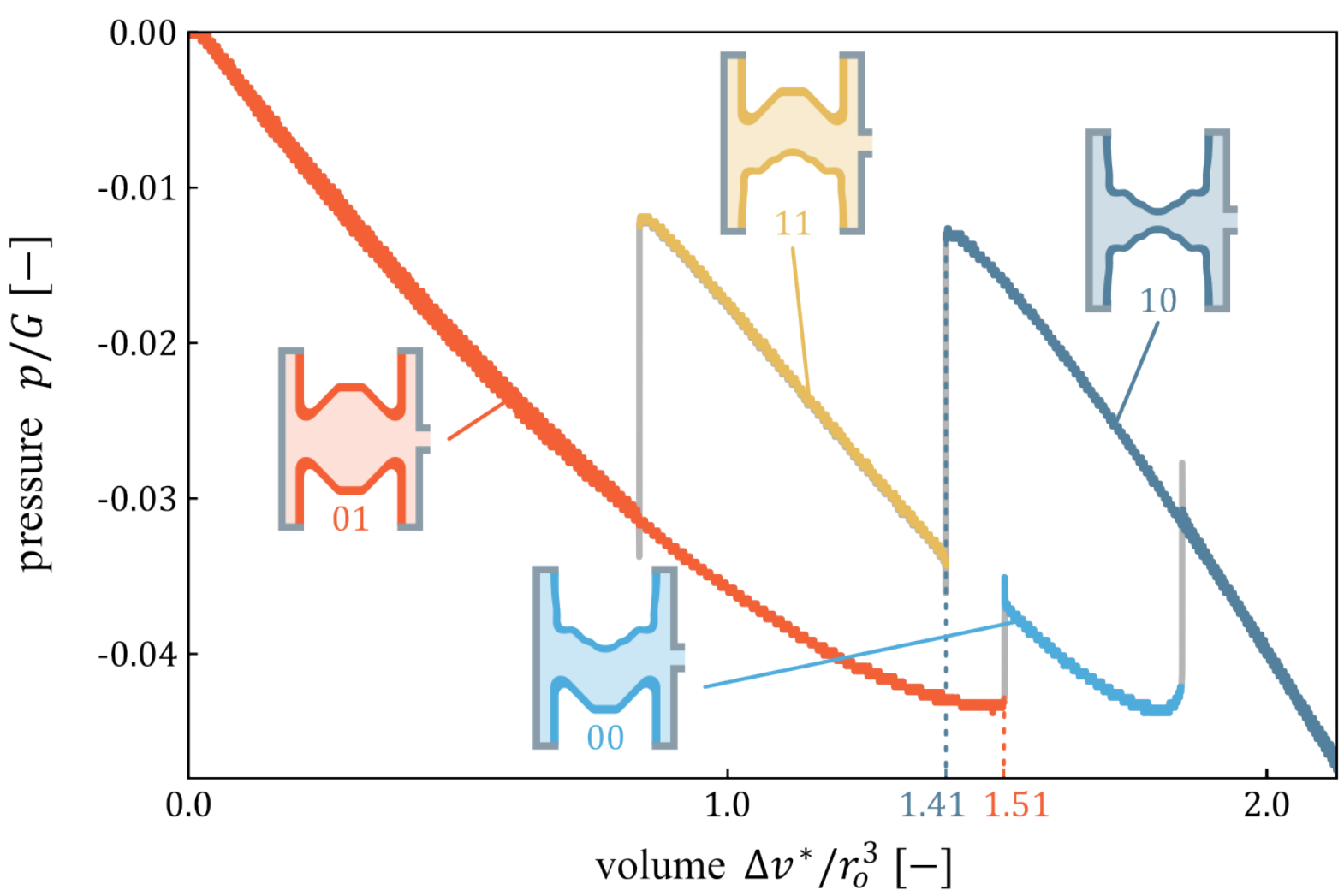

Fig. S3. Identification Stability Boundaries Volume-controlled pv-measurements obtained by extracting wtaer from the cavity between the parallel membranes at driving pressure ($p_{tot} = 0$). The curve is labeled with the corresponding collective states, indicating the stability regions for each configuration.

S3. Modeling

S3.1. Interaction under Coupled Actuation.

As explained in the main text, actuating a series configuration with volume control is conceptually equivalent to actuating a parallel configuration with pressure control, as both cases use the coupled variable to drive the system. This coupling leads to interaction between the connected inflatable structures.

Figure S4 illustrates this interaction using pv-curves of two generic inflatable structures: one linear and one nonlinear. The snapping events when actuating the different systems are marked by triangles. On the left, the membranes are actuated using volume control. In isolation, the nonlinear membrane exhibits snap-through behavior at the volumes $\Delta v_{\blacktriangleright}$ and $\Delta v_{\blacktriangleleft}$. For the parallel configuration, these snapping volumes remain unchanged ($\Delta v_{\blacktriangleright} = \Delta v^p_{\blacktriangleright}$ and $\Delta v_{\blacktriangleleft} = \Delta v^p_{\blacktriangleleft}$) since the structures are not volume-coupled. In contrast, for the series configuration, the snapping volumes increase ($\Delta v^s_{\blacktriangleright} > \Delta v_{\blacktriangleright}$ and $\Delta v^s_{\blacktriangleleft} > \Delta v_{\blacktriangleleft}$), indicating interaction due to the shared volume constraint.

On the right, the same systems are actuated using pressure control. In this case, the snapping pressures $p_{\blacktriangle}$ and $p_{\blacktriangledown}$ of the nonlinear structure remain unchanged in the series connection ($p_{\blacktriangle} = p^s_{\blacktriangle}$ and $p_{\blacktriangledown} = p^s_{\blacktriangledown}$) but shift in the parallel configuration ($p^p_{\blacktriangle} > p_{\blacktriangle}$ and $p^p_{\blacktriangledown} > p_{\blacktriangledown}$), again confirming interaction under the coupled variable.

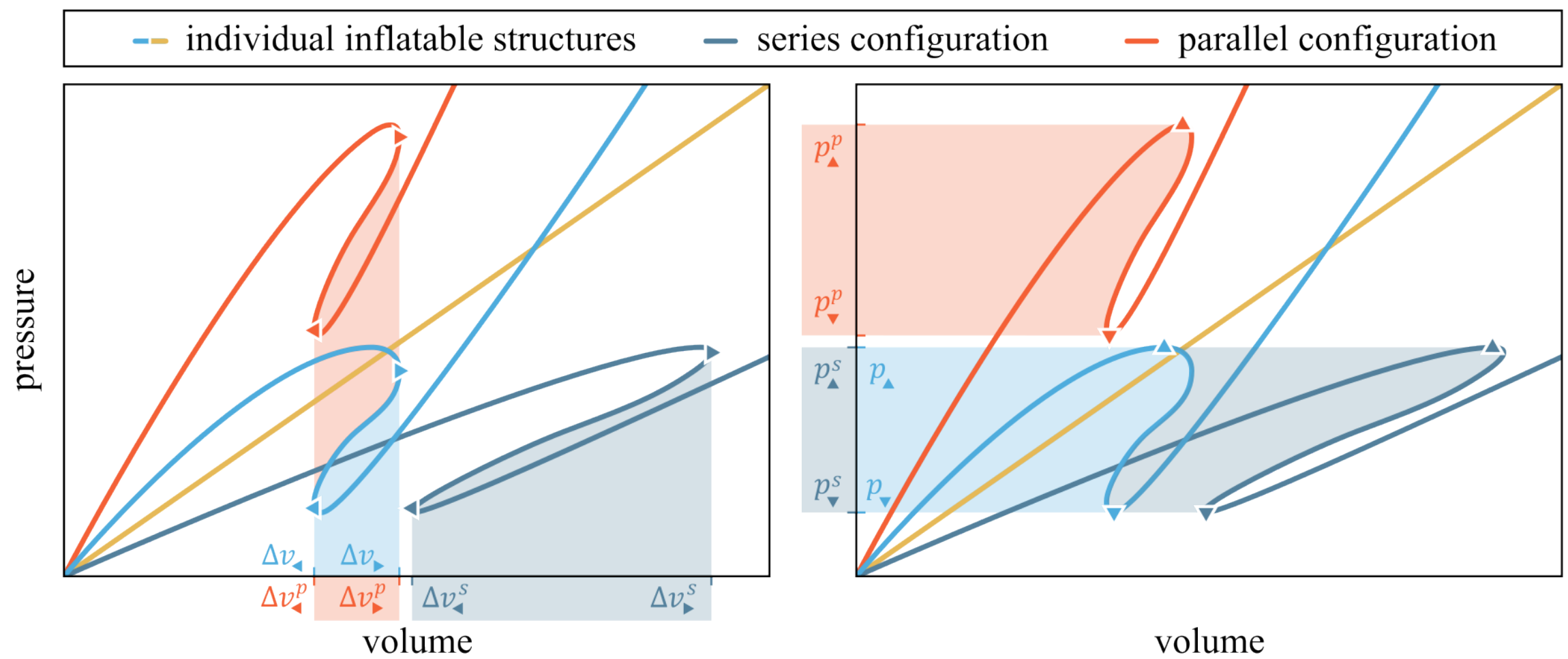

Fig. S4. Interactions Inflatable Structures Interaction dynamics under volume (left) and pressure (right) control for series and parallel connections. A generic nonlinear and linear membrane are shown individually and in combination. Under volume control, interaction occurs in the series setup (affected snapping volumes), while under pressure control, interaction is seen in the parallel setup (affected switching pressures).

S3.2. Emergence of Multiple Paths in Pressure-Volume Domain.

When connecting nonlinear inflatable structures, multiple paths can appear in the pressure-volume domain. Multiple paths arise when the individual structures have multiple values of the coupled variable (volume in a series connection or pressure in a parallel connection) for the same value of the uncoupled variable (pressure or volume, respectively) over an overlapping range of that uncoupled variable. Meaning that, for a series connection, this requires that both elements can occupy different volumes while sustaining the same pressure. For the parallel connection, this requires that both elements can occupy different pressures for the same volume change. In the particular case of the parallel fluidic hysterons studied in this paper, the emergence of multiple paths is illustrated in Figure S5.

By adjusting the presetting volume Δv^*, as described in the main text, the degree of overlap between the multi-valued regions of the individual parallel inflatable structures can be altered. When $\Delta v^* < 2\Delta v_{\blacktriangleleft}$, there is no volume at which both the blue and the orange hysterons can exhibit different pressures, and only a single path exists in the pv-domain.

At $\Delta v^* = 2\Delta v_{\blacktriangleleft}$ (middle top graph of Figure S5), the snap-back volumes of the blue and the orange hysterons coincide. From this presetting volume onward, a second path appears in the pv-domain (red path from the top middle graph to the bottom middle graph in Figure S5). In volumes where both paths coexist, both hysterons can exhibit multiple possible pressures at the same volume.

At $\Delta v^* = 2\Delta v_{\blacktriangleright}$ (middle bottom graph of Figure S5), the snap-forwards volumes of the blue and the orange hysterons coincide. Beyond this point, the system again has a single path in the pv-domain.

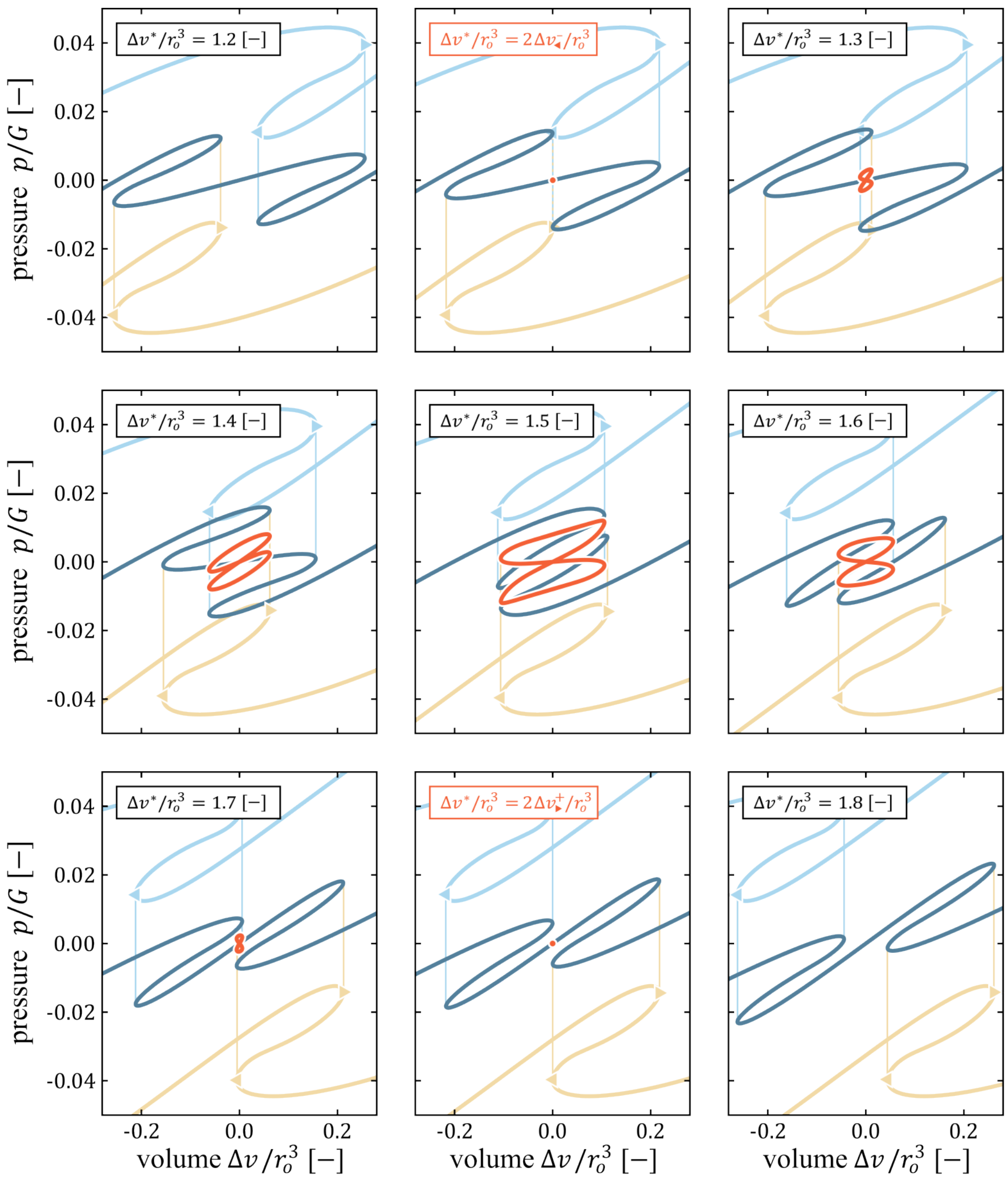

Fig. S5. Evolution of Pressure-Volume Curves for Parallel Fluidic Hysterons with Varying Presetting Volume Δv^* For each graph the pv-curves of the individual hysterons (blue and orange) are displayed as well as the pv-curve of the parallel system. The nine graphs illustrate how the total system evolves as $\Delta v^*/r_o^3$ increases from 1.2 to 1.8 $[-]$. At $\Delta v^* < 2\Delta v_{\blacktriangleleft}$ (top left), only a single path (grey) exists in the pv-domain. At $\Delta v^* = 2\Delta v_{\blacktriangleleft}$ (top middle), the snap-back volumes of both hysterons coincide, marking the onset of multiple paths in the pv-domain. Between this point and $\Delta v^* = 2\Delta v_{\blacktriangleright}$ (bottom middle), two distinct paths (grey and red) coexist. At $\Delta v^* = 2\Delta v_{\blacktriangleright}$, the snap-forward volumes coincide, after which only a single path remains (bottom right)

S3.3. Analytical Expressions Switching Pressures.

To construct the analytical expressions for the switching pressures in function of the volume preset Δv^*, the constructed model and the approach of van Liu et al. is used (2). This approach suggests that for $P_i^+\{S\}$ ($P_i^-\{S\}$) the ith hysteron will switch states, thus is reaching its up-switching pressure p_i^+ (down-switching pressure p_i^-). To find $P_i^{\pm}\{S\}(\Delta v^*)$, the equations of the parallel system and the model equations of the membranes in the collective state $S = \{s_1, s_2\}$ are used to first find $P_i\{S\}(p_i, \Delta v^*)$:

$$\begin{cases} p_{tot} = p_1(\Delta v_1) + p_2(\Delta v_2) \\ \Delta v_1(p_1) = \Delta v_2(p_2) + \Delta v^* \end{cases} \quad \text{[S1]}$$

$$
\begin{aligned}
&\text{for } s_1 = 0: \quad p_1 = -p_1^+ \left[\left(\frac{\Delta v_1}{\Delta v_1^+} \right)^2 - 2 \frac{\Delta v_1}{\Delta v_1^+} \right] \\
&\text{for } s_1 = 1: \quad p_1 = \frac{p_1^+ - p_1^-}{d_1} (\Delta v_1 - \Delta v_1^-) + p_1^-
\end{aligned}
\tag{S2}
$$

$$
\begin{aligned}
&\text{for } s_2 = 0: \quad p_2 = \frac{p_2^+ - p_2^-}{d_2} (\Delta v_2 - \Delta v_2^+) + p_2^+ \\
&\text{for } s_2 = 1: \quad p_2 = -p_2^- \left[\left(\frac{\Delta v_2}{\Delta v_2^-} \right)^2 - 2 \frac{\Delta v_2}{\Delta v_2^-} \right]
\end{aligned}
\tag{S3}
$$

Using these expressions it can be stated that $P_i^{\pm}\{S\}(\Delta v^*) = P_i\{S\}(p_i = p_i^{\pm}, \Delta v^*)$. This results in eight distinct analytical expression in function of ten parameters (p_1^+, Δv_1^+, p_1^-, Δv_1^-, d_1, p_2^+, Δv_2^+, p_2^-, Δv_2^- and d_2):

$$
\begin{aligned}
P_1^+\{00\}(\Delta v^*) &= \; p_1^+ + p_2^+ + \frac{p_2^+ - p_2^-}{d_2} (\Delta v_1^+ - \Delta v_2^+ - \Delta v^*) \\
P_1^+\{01\}(\Delta v^*) &= \; p_1^+ + p_2^- - p_2^- \left[1 - \frac{\Delta v_1^+ - \Delta v^*}{\Delta v_2^-} \right]^2 \\
P_1^-\{10\}(\Delta v^*) &= \; p_1^- + p_2^+ + \frac{p_2^+ - p_2^-}{d_2} (\Delta v_1^- - \Delta v_2^+ - \Delta v^*) \\
P_1^-\{11\}(\Delta v^*) &= \; p_1^- + p_2^- - p_2^- \left[1 - \frac{\Delta v_1^- - \Delta v^*}{\Delta v_2^-} \right]^2 \\
P_2^+\{00\}(\Delta v^*) &= \; p_2^+ + p_1^+ - p_1^+ \left[1 - \frac{\Delta v_2^+ + \Delta v^*}{\Delta v_1^+} \right]^2 \\
P_2^-\{01\}(\Delta v^*) &= \; p_2^- + p_1^+ - p_1^+ \left[1 - \frac{\Delta v_2^- + \Delta v^*}{\Delta v_1^+} \right]^2 \\
P_2^+\{10\}(\Delta v^*) &= \; {}_1^- + p_2^+ + \frac{p_1^+ - p_1^-}{d_1} (\Delta v_2^+ - \Delta v_1^- + \Delta v^*) \\
P_2^-\{11\}(\Delta v^*) &= \; p_2^- + p_1^- + \frac{p_1^+ - p_1^-}{d_1} (\Delta v_2^- - \Delta v_1^- + \Delta v^*)
\end{aligned}
\tag{S4}
$$

Because the following is true for two identical, but flipped membranes:

$$
p_1^+ = -p_2^- \quad , \quad p_1^- = -p_2^+ \quad , \quad \Delta v_1^+ = -\Delta v_2^- \quad , \quad \Delta v_1^- = -\Delta v_2^+ \quad \& \quad d_1 = d_2 \tag{S5}
$$

(as can be seen in Figure S6), the equations can be simplified more to be only a function of $p^+, p^-, \Delta v^+, \Delta v^-$ and d. The results of this simplification can be found in Equations 8 and 9 of the main text.

The volume controlled measured pressure-volume data, done like reported in the Supporting Information by Van Raemdonck et al (1) and shown in Figure S6, of the two membranes is used:

$$
\begin{array}{ccccccccc}
p^+ & = & p_1^+ & = & -p_2^- & = & \frac{p_{1,meas}^+ - p_{2,meas}^-}{2} & = & 0.271 \text{ bar} \\
p^- & = & p_1^- & = & -p_2^+ & = & \frac{p_{1,meas}^- - p_{2,meas}^+}{2} & = & 0.0836 \text{ bar} \\
\Delta v^+ & = & \Delta v_1^+ & = & -\Delta v_2^- & = & \frac{\Delta v_{1,meas}^+ - \Delta v_{2,meas}^-}{2} & = & 0.791 \text{ ml} \\
\Delta v^- & = & \Delta v_1^- & = & -\Delta v_2^+ & = & \frac{\Delta v_{1,meas}^- - \Delta v_{2,meas}^+}{2} & = & 0.706 \text{ ml} \\
d & = & d_1 & = & d_2 & = & \frac{d_{1,meas} + d_{2,meas}}{2} & = & 0.357 \text{ ml}
\end{array}
\tag{S6}
$$

The constructed expressions of $P_i\{S\}(p_i, \Delta v^*)$ are used to get to the final result:

$$
\begin{aligned}
P_1^+\{S\}(\Delta v^*) &= P_1\{S\}(p_1 = p^+, \Delta v^*) \\
P_1^-\{S\}(\Delta v^*) &= P_1\{S\}(p_1 = p^-, \Delta v^*) \\
P_2^+\{S\}(\Delta v^*) &= P_2\{S\}(p_2 = -p^-, \Delta v^*) \\
P_2^-\{S\}(\Delta v^*) &= P_2\{S\}(p_2 = -p^+, \Delta v^*)
\end{aligned}
\tag{S7}
$$

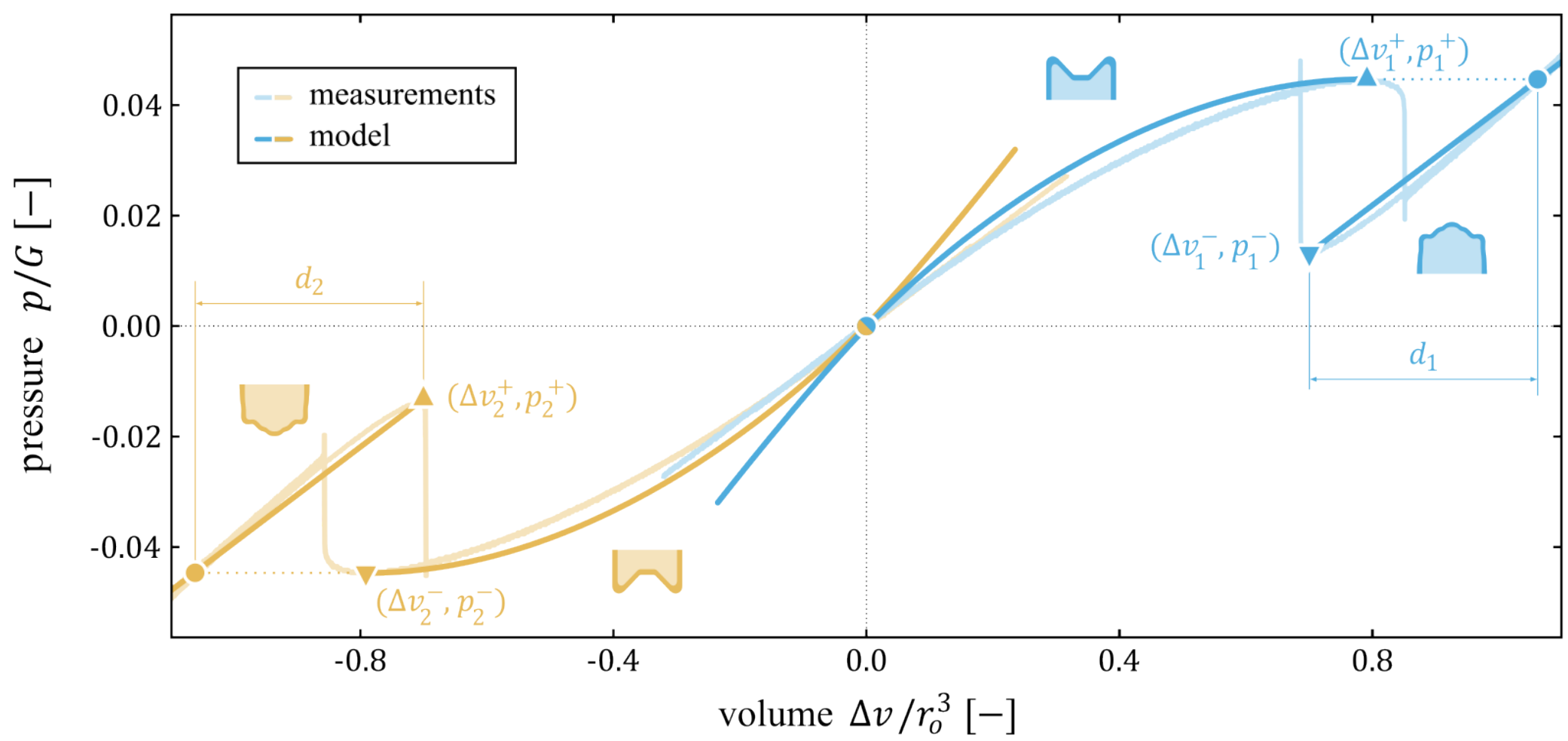

Fig. S6. Model Fluidic Hysterons Parabolic-linear models fitted to the pv-measurements of the actuated (blue) and responsive (orange) membranes, used to represent their individual hysteretic behavior.

To analyse the difference between the two-hysteron symmetric case and the two-hysteron asymetric case, switching pressures are calculated for two identical hysterons with data:

$$p^+ = 0.271 \text{ bar} \quad , \quad p^- = 0.0836 \text{ bar} \quad , \quad \Delta v^+ = 0.791 \text{ ml} \quad , \quad \Delta v^- = 0.706 \text{ ml} \quad \& \quad d = 0.357 \text{ ml} \qquad \text{[S8]}$$

as used in the main text. For the two-hysteron asymmetric case, the second hysteron has been replaced by a hysteron with the following data:

$$p_2^+ = -0.0732 \text{ bar} \quad , \quad p_2^- = -0.293 \text{ bar} \quad , \quad \Delta v_2^+ = -0.700 \text{ ml} \quad , \quad \Delta v_2^- = -0.800 \text{ ml} \quad \& \quad d_2 = 0.430 \text{ ml} \qquad \text{[S9]}$$

The comparison for the switching pressures of these two cases can be found in Figure S7. Because of the asymmetry in the bottom graph, two additional state-diagram regions appear (labeled (a') and (e')). In these regions the system exhibits an avalanche in one direction of pressure change (increasing pressure for (a') and decreasing pressure for (e')), but not in the opposite direction. In principle, such zones can also occur in an experimentally symmetric system, because no two fluidic hysterons are ever perfectly identical due to fabrication differences. However, these deviations are usually small, making it practically very unlikely for the system to operate within these additional zones.

S3.4. Model Comparisson for the Fluidic Hysteron.

In the work of Liu et al. (2), a linear-linear approximation was used to model a mechanical hysteron in the force-displacement domain. The same approach was applied here to the fluidic hysteron studied in this paper, as shown in the top left graph of Figure S8. Because the model consists of two parallel lines, only four data points from the pv-measurements are required: p^+, Δv^+, p^-, and Δv^-. The top right graph compares the analytical transition pressures obtained from this model to the experimental results. Although the model predicts five distinct zones, the boundaries between zones (a) and (b) and between zones (d) and (e) occur at larger preset volumes Δv^* than observed experimentally.

To improve the accuracy of the approximation, a modified model using two non-parallel lines was tested. This model requires one additional data point, denoted d, which represents the offset in volume of the transition snap point compared to the snap-down volume Δv^-, i.e. $\Delta v^- + d$. As shown in the middle left panel of Figure S8, this adjustment results in a better fit of the state 1 branch of the fluidic hysteron, which in turn leads to an improved prediction of the boundary between zones (d) and (e) (see middle right graph Figure S8).

However, the other zone boundaries remain unaffected, and the first half of the measurements is still not well captured. To address this, the state 0 branch of the hysteron was updated from a linear to a parabolic approximation. This modification maintains computational simplicity and does not require additional data points, while providing a closer agreement between the analytical expressions and the experimental data, as shown in the bottom right panel of Figure S8.

S3.5. Construction State Diagrams.

The state diagrams shown in Figures 3**B**, 4**B**, and 5**C** were constructed following the approach described by Teunisse et al. (3). These diagrams depend on the relative ordering of the switching pressures; whenever this order changes, a different zone with distinct state stability emerges. For each zone in which the order of switching pressures remains unchanged, the corresponding stability ranges are indicated. An example for zone (b) is shown in Figure S9. Once these ranges are identified,

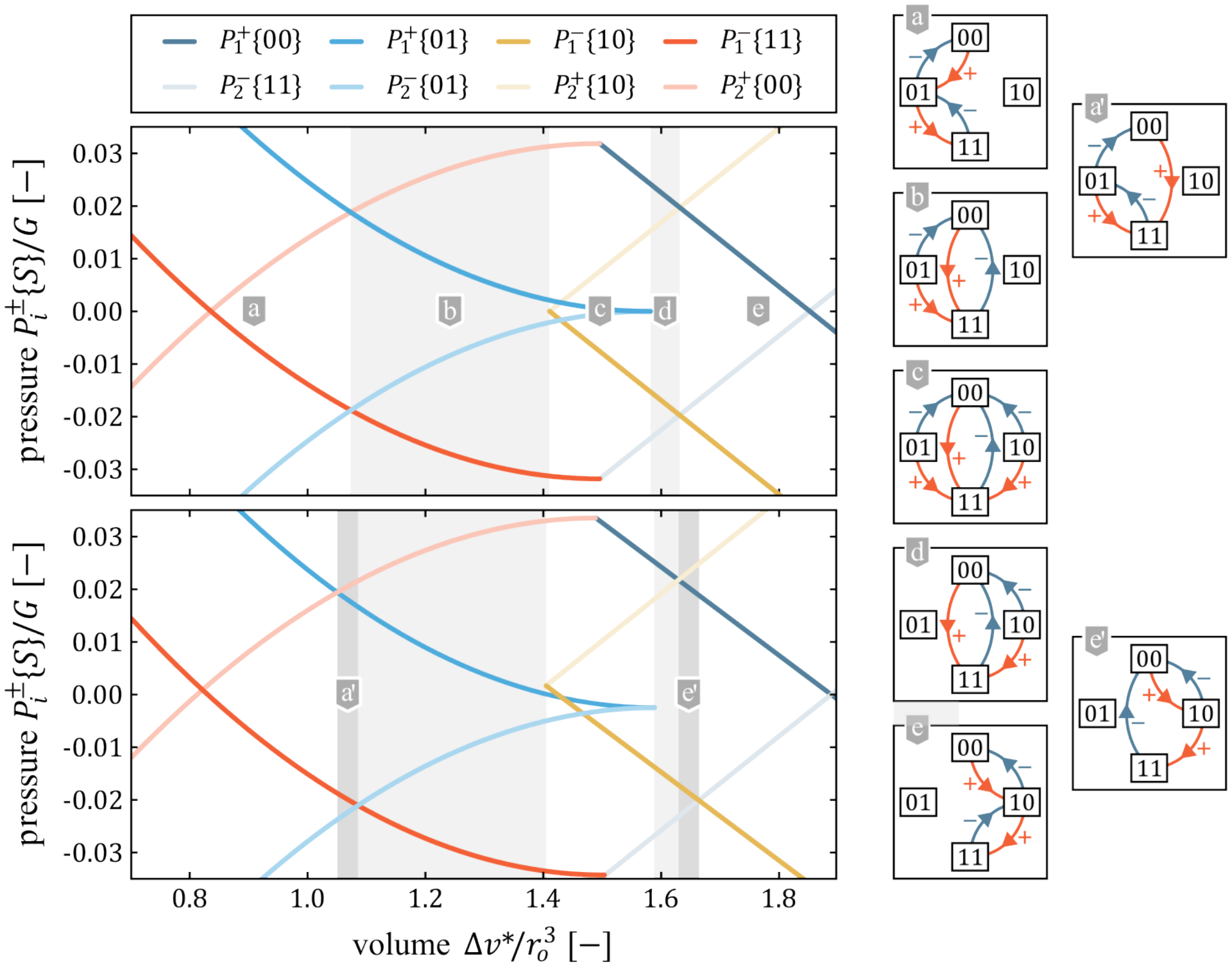

Fig. S7. Switching pressures $P_i^{\pm}\{S\}$ for symmetric and asymmetric parallel hysterons. This figure compares the switching pressures $P_i^{\pm}\{S\}$ as a function of the preset volume Δv^* for two parallel-connected fluidic hysterons. The top panel shows the symmetric case, in which the hysterons are identical but mirrored. The bottom panel shows an asymmetric case, where the two hysterons have different pressure–volume characteristics and therefore distinct hysteron parameters (p^+, Δv^+, p^-, Δv^- and d). While the same fundamental state diagrams emerge in both cases, the asymmetric configuration gives rise to two additional state diagrams, labeled (a') and (e').

the system is initialized in each stable state. By increasing or decreasing the driving pressure P, the resulting state transitions can be mapped, enabling the complete construction of the state diagram.

S3.6. Analytical Derivation of State-Diagram Zone Boundaries.

In this section, analytical expressions are derived for the locations of the boundaries between the different state-diagram zones shown in Figure 4**B** of the main text. These boundary locations are expressed as functions of the hysteron parameters p^+, Δv^+, p^-, Δv^- and d. Transitions between zones occur either when the ordering of key switching pressures changes or when the stability range of a particular state vanishes. Each of the four boundaries separating the five zones is derived below.

Boundary (a)-(b). In zone (a), $P_2^+\{00\} < P_1^+\{01\}$ (equivalently, $P_2^-\{01\} < P_1^-\{11\}$) is satisfied. As the preset volume Δv^* increases, this ordering reverses, marking the transition to zone (b). The boundary is therefore defined by

$$P_2^+\{00\} = P_1^+\{01\} \tag{S10}$$

By symmetry, this condition can be rewritten as

$$\begin{gathered} -P_1^-\{11\} = P_1^+\{01\} \\ -p^- + p^+ - p^+\left[1 + \frac{\Delta v^- - \Delta v^*}{\Delta v^+}\right]^2 = p^+\left[2 - \frac{\Delta v^*}{\Delta v^+}\right]^2 \end{gathered} \tag{S11}$$

Solving for Δv^* yields two intersection points between the corresponding parabolic curves:

$$\Delta v^* = \frac{\Delta v^- + 3\Delta v^+}{2} \pm \frac{1}{2}\sqrt{-(\Delta v^-)^2 + 2\Delta v^-\Delta v^+ + \left(1 - 2\frac{p^-}{p^+}\right)(\Delta v^+)^2} \tag{S12}$$

The physically relevant boundary corresponds to the first intersection as Δv^* increases. The location of the (a)-(b) boundary is therefore

$$\Delta v^* = \frac{\Delta v^- + 3\Delta v^+}{2} - \frac{1}{2}\sqrt{-(\Delta v^-)^2 + 2\Delta v^-\Delta v^+ + \left(1 - 2\frac{p^-}{p^+}\right)(\Delta v^+)^2} \tag{S13}$$

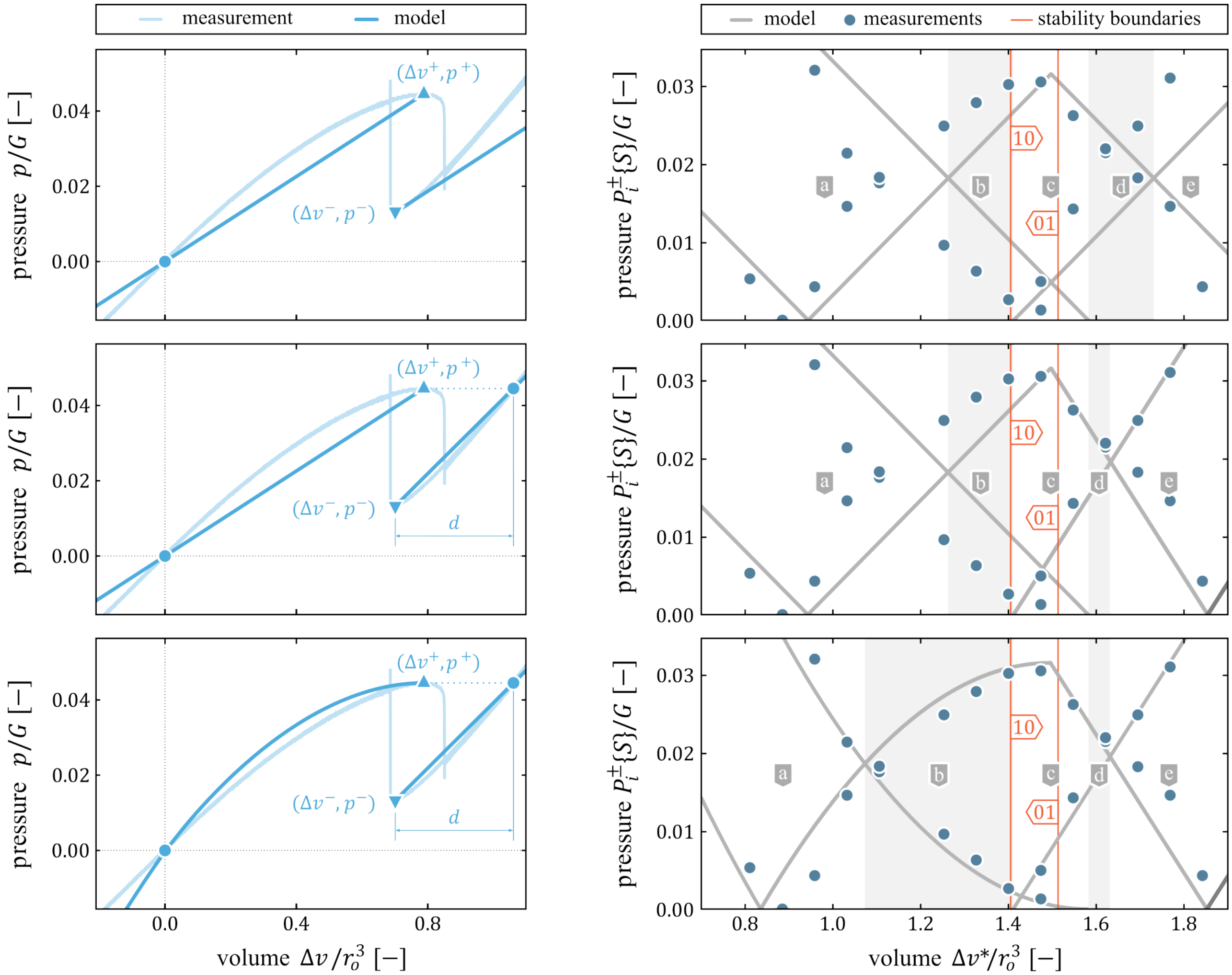

Fig. S8. Comparison of Analytical Models for the Fluidic Hysteron Each row presents a different analytical model for the fluidic hysteron. The left column shows the proposed models fitted to the volume-controlled measurements of the blue fluidic hysteron. The right column compares the analytically predicted transition pressures from each model with the experimental results. Top row: Linear–linear approximation following Liu et al. (2), based on four data points (p^+, Δv^+, p^-, Δv^-). Middle row: Modified model with two non-parallel lines, including an additional data point d to better approximate the state 1 branch. Bottom row: Linear–parabolic model, where the state 0 branch is replaced by a parabolic approximation. This model provides the best agreement with the experiments and is the one used in the main text.

Boundary (b)-(c). In zone (b), avalanches occur and $\{01\}$ is the only Garden-of-Eden state. At a sufficiently large preset volume, acquires $\{01\}$ a stability range. This occurs when the switching pressure $P_1^-\{10\}$ (equivalently, $P_2^+\{10\}$) crosses zero:

$$P_1^-\{10\} = \frac{(p^+ - p^-)}{d}(2\Delta v^- - \Delta v^*) = 0 \qquad \text{[S14]}$$

Hence, the (b)-(c) boundary is located at

$$\Delta v^* = 2\Delta v^- \qquad \text{[S15]}$$

Boundary (c)-(d). Within zone (c), both $\{01\}$ and $\{10\}$ are stable. As Δv^* increases, the pressure stability range of $\{10\}$ shrinks and eventually vanishes. This occurs when

$$P_1^+\{01\} = p^+\left(2 - \frac{\Delta v^*}{\Delta v^+}\right)^2 = 0 \qquad \text{[S16]}$$

Therefore, the location of the (c)-(d) boundary is given by

$$\Delta v^* = 2\Delta v^+ \qquad \text{[S17]}$$

Boundary (d)-(e). Zone (d) is characterized by avalanches behavior, which disappear when $P_2^+\{10\} > P_1^+\{00\}$ (equivalently, $P_2^-\{11\} > P_1^-\{10\}$). The boundary is defined by

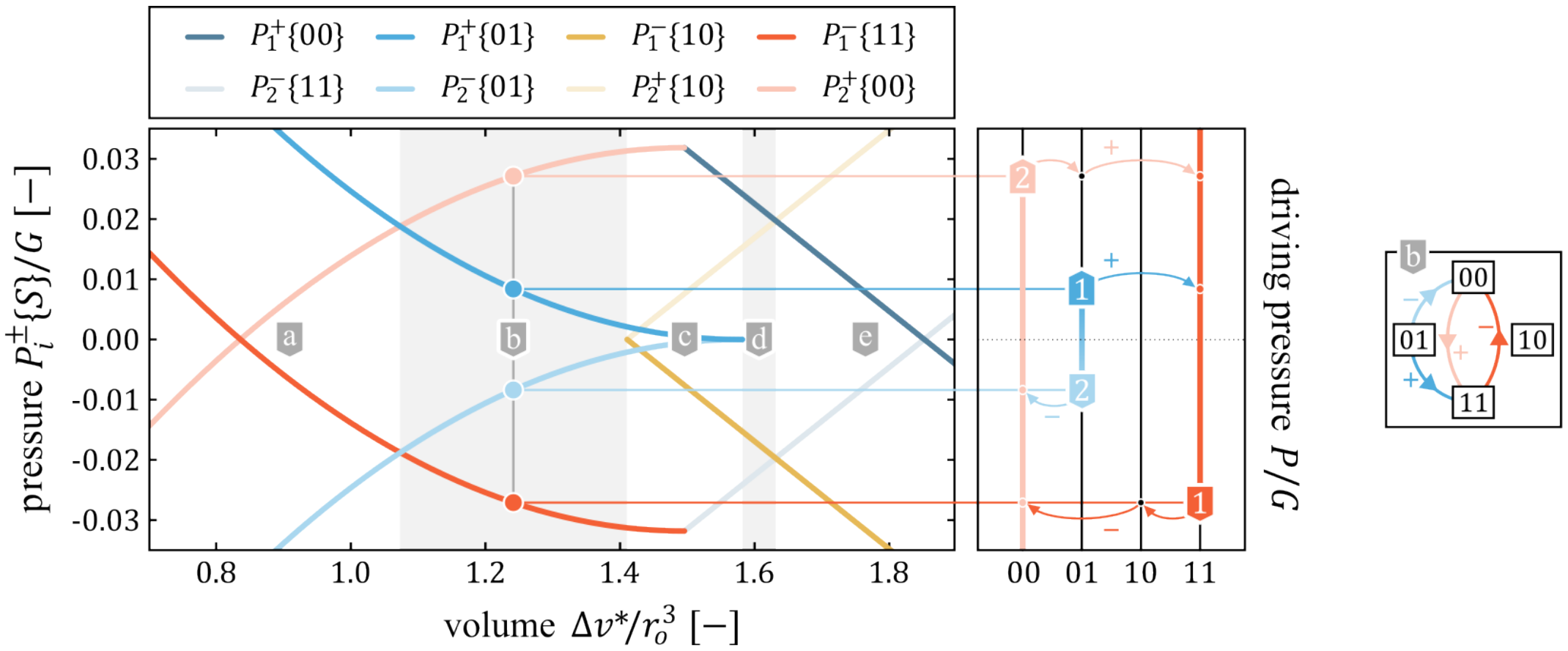

Fig. S9. Construction of the State Diagram. Illustration of the state diagram construction for zone (b). The central panel displays the pressure stability ranges for the four collective states as a function of the driving pressure P. The system is theoretically initialized in each stable state. Starting from the $\{00\}$ state, increasing P causes a transition to the $\{01\}$ state. However, since $\{01\}$ is unstable at $P = P_2^+\{00\}$, the system undergoes an *avalanche* transition to the $\{11\}$ state. Similarly, when initialized in the $\{11\}$ state, decreasing P leads to an *avalanche* back to the $\{00\}$ state via the always-unstable $\{10\}$ state. If the system starts in the $\{01\}$ state, increasing (decreasing) P results in a transition to the $\{11\}$ ($\{00\}$) state.

$$P_2^+\{10\} = P_1^+\{00\} \quad \text{[S18]}$$

Using symmetry

$$\begin{gathered}-P_1^-\{10\} = P_1^+\{00\} \\ -\frac{p^+ - p^-}{d}(2\Delta v^- - \Delta v^*) = (p^+ - p^-)\left[1 + \frac{\Delta v^+ + \Delta v^- - \Delta v^*}{d}\right]\end{gathered} \quad \text{[S19]}$$

The location of the (d)-(e) boundary thus becomes:

$$\Delta v^* = \frac{3\Delta v^- + \Delta v^+ + d}{2} \quad \text{[S20]}$$

Numerical Boundary Locations. These boundary locations can now be evaluated for the symmetric system studied in the main text by using the parameters stated in Equation S6:

$$(a)-(b): \Delta v^* = 1.08 \text{ ml} \quad , \quad (b)-(c): \Delta v^* = 1.41 \text{ ml} \quad , \quad (c)-(d): \Delta v^* = 1.58 \text{ ml} \quad \& \quad (d)-(e): \Delta v^* = 1.63 \text{ ml} \quad \text{[S21]}$$

Parameter Dependence and Robustness. The analytical expressions above enable systematic investigation of how hysteron parameters influence the extent of each zone. The (b)–(c) and (c)–(d) boundaries depend exclusively on Δv^- and Δv^+, respectively, each shifting linearly with two times the rate when changing the preset volume Δv^*. The (d)–(e) boundary depends on all volume parameters and shifts 1.5 times as fast with Δv^- and half as fast with Δv^+ and d. These relative sensitivities are summarized visually in Figure S10, which illustrates how variations in the hysteron parameters redistribute the widths of the different state-diagram zones.

The dependence of the (a)-(b) boundary (Equation S13) is less straightforward. To approximate the rate of change for these different parameters, the following dimensionless parameters are introduced:

$$\alpha = \frac{\Delta v^-}{\Delta v^+} \quad \& \quad \beta = \frac{p^-}{p^+} \quad \text{[S22]}$$

Equation S13 can be rewritten:

$$\Delta v^* = \frac{\Delta v^+}{2}\left[3 + \frac{1}{\alpha} - \sqrt{1 + \frac{2\alpha - 1}{\alpha^2} - 2\beta}\right] \quad \text{[S23]}$$

or equivalently,

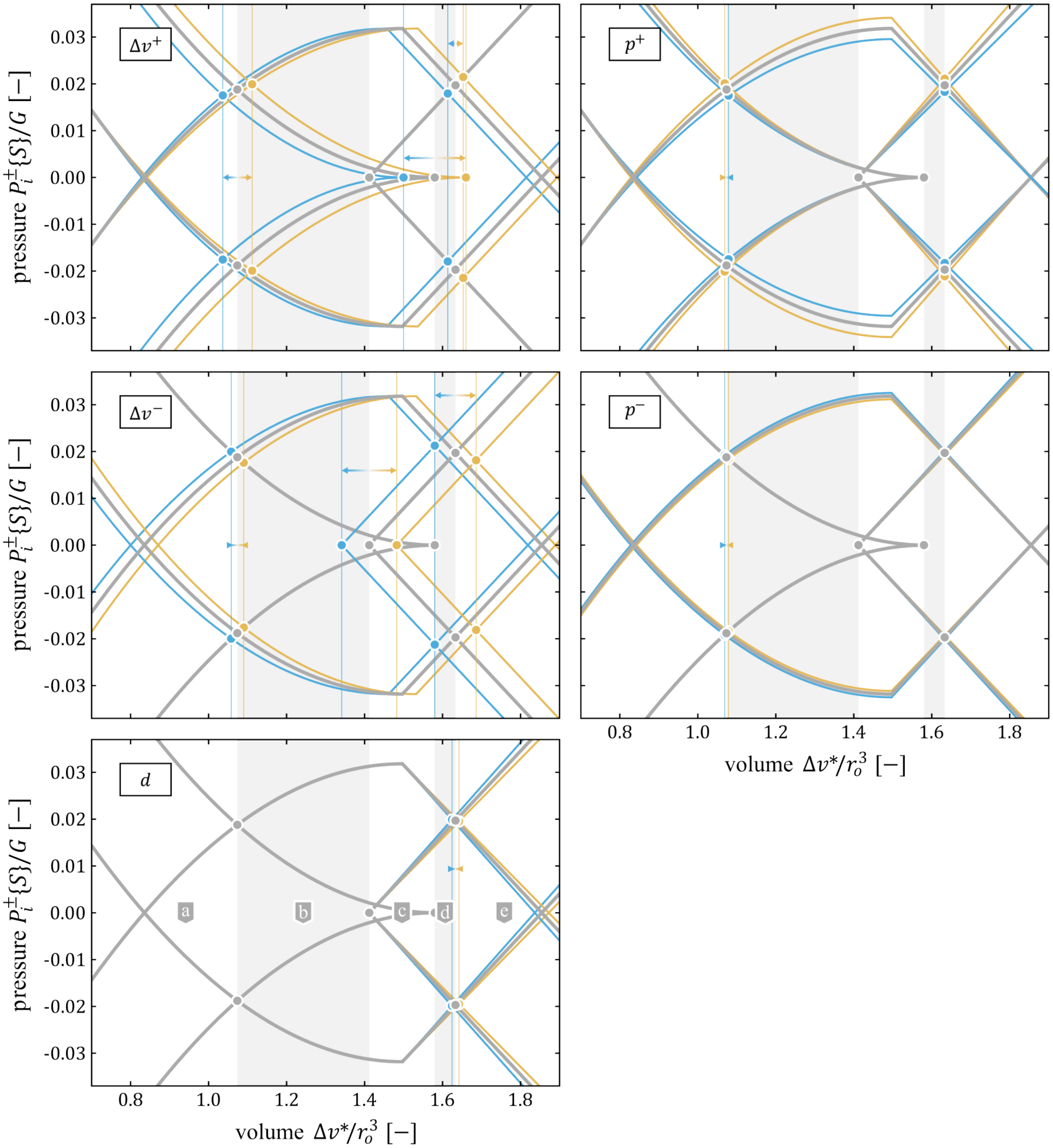

Fig. S10. Visual Sensitivity Analyses Zone Boundary Locations. Visualization of how variations in individual hysteron parameters affect the locations of the state-diagram zone boundaries shown in Figure 4**B**. In each panel, one hysteron parameter (p^+, Δv^+, p^-, Δv^-, or d) is varied independently by $\pm 5\%$, while all other parameters are held constant and the switching pressures are recalculated. The labels for each zone ((a) through (e)) are indicated in the bottom left graph. Gray curves indicate the switching pressures for the nominal parameter set. Orange and blue curves correspond to a $+5\%$ and -5% variation of the indicated parameter, respectively. Arrows indicate both the direction and relative magnitude of the corresponding zone-boundary shifts for increasing or decreasing parameter values.

$$\Delta v^* = \frac{\Delta v^-}{2}\left[1 + 3\alpha - \alpha\sqrt{1 + \frac{2\alpha - 1}{\alpha^2} - 2\beta}\right] \qquad \text{[S24]}$$

For inflatable hysterons similar to those studied here, practical fabrication constraints typically satisfy $0.8 < \alpha < 1.2$ and $0.2 < \beta < 0.3$. Within these ranges, the (a)–(b) boundary varies between 1.29 – 1.55 times as fast as Δv^+ and 1.20 - 1.60 times as fast as Δv^-. The influence of p^+ and p^- enters only through ratio β and is comparatively weak.

Taken together, these scaling relations show that the boundaries delimiting zone (c) are significantly more sensitive to variations in the snapping-up and snapping-down volumes than the two outer boundaries. As a result, sufficiently small values of Δv^- can cause zone (b) to vanish, while large values of Δv^+ or small values of Δv^- can eliminate zone (d). Both effects are

visualized in Figure S10, where the redistribution of zone widths under parameter variation is shown. The same figure also illustrates that changes in the snapping-up and snapping-down pressures primarily act to scale the switching pressures, with the exception of their weaker, indirect influence on the (a)–(b) boundary discussed above.

In summary, this analysis shows that the qualitative topology of the state diagram is preserved under moderate parameter variations, the inner zone boundaries are more sensitive than the outer boundaries, and variations in the pressure parameters primarily rescale the switching pressures rather than shifting the zone boundaries.

Video S1. Surge Protector Demonstrator. Experimental demonstration of a asymmetric parallel-connected fluidic two-hysteron system exhibiting tunable response under pressure control. The system consists of two membranes coupled through water, actuated via pressure input applied to the right (red) membrane. The top panel shows the applied pressure signal as a function of time. During normal operation, the input pressure oscillates between p_{min} and p_{max}. For an initial preset volume of $\Delta v_1^* = 0.922$ ml, this oscillatory input causes periodic snapping of the left (blue) membrane, making the system switch between states $\{00\}$ and $\{01\}$. A pressure spike of amplitude p_{spike} at $t = 10$ s drives both membranes to the left, setting the system into the $\{11\}$ state. After the spike, the input pressure returns to the oscillatory range $[p_{min}, p_{max}]$, after which the system spontaneously recovers oscillatory behavior.

The preset volume is then increased to $\Delta v_2^* = 0.996$ ml by adjusting the coupling volume. Under the same pressure input profile, including the identical spike p_{spike}, the system becomes trapped in the $\{11\}$ state, suppressing further oscillations. A brief manual perturbation at $t = 15.5$ s resets the system to $\{00\}$, after which oscillatory switching resumes. This video illustrates how the preset volume selects distinct regimes, enabling surge-protection-like behavior.

References

1. Bert Van Raemdonck, Edoardo Milana, Michael De Volder, Dominiek Reynaerts, and Benjamin Gorissen. Nonlinear inflatable actuators for distributed control in soft robots. *Advanced Materials*, 35(35):2301487, 2023. . URL https://advanced.onlinelibrary.wiley.com/doi/abs/10.1002/adma.202301487.
2. Jingran Liu, Margot Teunisse, George Korovin, Ivo R. Vermaire, Lishuai Jin, Hadrien Bense, and Martin van Hecke. Controlled pathways and sequential information processing in serially coupled mechanical hysterons. *Proceedings of the National Academy of Sciences*, 121(22):e2308414121, 2024. . URL https://www.pnas.org/doi/abs/10.1073/pnas.2308414121.
3. Margot H. Teunisse and Martin van Hecke. Transition graphs of interacting hysterons: Structure, design, organization and statistics. 2024. URL https://arxiv.org/abs/2404.11344.
